# Immune Differentiation Regulator p100 Tunes NF-κB Responses to TNF

**DOI:** 10.3389/fimmu.2019.00997

**Published:** 2019-05-07

**Authors:** Budhaditya Chatterjee, Payel Roy, Uday Aditya Sarkar, Mingming Zhao, Yashika Ratra, Amit Singh, Meenakshi Chawla, Supriyo De, James Gomes, Ranjan Sen, Soumen Basak

**Affiliations:** ^1^Systems Immunology Laboratory, National Institute of Immunology, New Delhi, India; ^2^Kusuma School of Biological Sciences, Indian Institute of Technology Delhi, New Delhi, India; ^3^Gene Regulation Section, Laboratory of Molecular Biology and Immunology, National Institute on Aging, Baltimore, MD, United States; ^4^Laboratory of Genetics and Genomics, National Institute on Aging, Baltimore, MD, United States

**Keywords:** TNF, pulsatile, NF-kappaB, p100, temporal control, gene-expression specificity

## Abstract

Tumor necrosis factor (TNF) is a pleiotropic cytokine whose primary physiological function involves coordinating inflammatory and adaptive immune responses. However, uncontrolled TNF signaling causes aberrant inflammation and has been implicated in several human ailments. Therefore, an understanding of the molecular mechanisms underlying dynamical and gene controls of TNF signaling bear significance for human health. As such, TNF engages the canonical nuclear factor kappa B (NF-κB) pathway to activate RelA:p50 heterodimers, which induce expression of specific immune response genes. Brief and chronic TNF stimulation produces transient and long-lasting NF-κB activities, respectively. Negative feedback regulators of the canonical pathway, including IκBα, are thought to ensure transient RelA:p50 responses to short-lived TNF signals. The non-canonical NF-κB pathway mediates RelB activity during immune differentiation involving p100. We uncovered an unexpected role of p100 in TNF signaling. Brief TNF stimulation of p100-deficient cells triggered an additional late NF-κB activity consisting of RelB:p50 heterodimers, which modified the TNF-induced gene-expression program. In p100-deficient cells subjected to brief TNF stimulation, RelB:p50 not only sustained the expression of a subset of RelA-target immune response genes but also activated additional genes that were not normally induced by TNF in WT mouse embryonic fibroblasts (MEFs) and were related to immune differentiation and metabolic processes. Despite this RelB-mediated distinct gene control, however, RelA and RelB bound to mostly overlapping chromatin sites in p100-deficient cells. Repeated TNF pulses strengthened this RelB:p50 activity, which was supported by NF-κB-driven RelB synthesis. Finally, brief TNF stimulation elicited late-acting expressions of NF-κB target pro-survival genes in p100-deficient myeloma cells. In sum, our study suggests that the immune-differentiation regulator p100 enforces specificity of TNF signaling and that varied p100 levels may provide for modifying TNF responses in diverse physiological and pathological settings.

## Introduction

Tumor necrosis factor (TNF) is a pleiotropic cytokine whose primary physiological function involves coordinating innate and adaptive immune responses (1). TNF engages the canonical NF-κB pathway to activate RelA:p50 NF-κB heterodimers that are sequestered in the cytoplasm of unstimulated cells by inhibitor of κB (IκB) α, β, and ε proteins (2). In the canonical pathway, TNF treatment induces the IκB kinase (IKK) complex consisting of NEMO and IKK2 (or IKKβ), which phosphorylates IκBs leading to their degradation and nuclear translocation of RelA:p50. In the nucleus, RelA:p50 mediate the expression of specific pro-inflammatory and immune response genes.

Typically, TNF briefly stimulates tissue resident cells due to its short half-life *in vivo* (3). Previous studies demonstrated that the NF-κB system, in fact, distinguishes between brief and chronic TNF signals for a wide range of TNF concentrations (4–6). Brief TNF stimulation induces a transient RelA:p50 activity peak persisting in the nucleus for about an hour. In contrast, chronic TNF stimulation triggers an additional second wave of protracted RelA:p50 activity, which lasts in the nucleus for more than 8 h. This late RelA:p50 activity displays oscillatory behavior at single-cell resolution (7). Importantly, chronic TNF treatment activates a distinct set of late-acting NF-κB target genes that are not induced upon brief TNF stimulation (4, 8). Regardless of the duration of TNF treatment, RelA:p50 induce rapid synthesis of the inhibitors of the canonical pathway, including IκBα, IκBε, and A20 (9, 10). A series of elegant studies suggested that coordinated functioning of these negative feedback regulators determines dynamical RelA:p50 responses to time-varied TNF inputs (6, 11–13). It is thought that RelA:p50 regulation by the canonical NF-κB pathway largely provides for distinct transcriptional outputs to brief and chronic TNF stimulations (14). On the other hand, deregulated TNF signaling has been implicated in several human ailments, including inflammatory bowel disorders and neoplastic diseases (1).

The non-canonical NF-κB pathway mediates a separate RelB-containing NF-κB activity. In resting cells, p100 encoded by *Nfkb2* retains RelB and other NF-κB proteins in the cytoplasm (15). Non-canonical signaling induced by B-cell activating factor (BAFF) or lymphotoxin α_1_β_2_ (LTα_1_β_2_) activates a complex consisting of NF-κB inducing kinase (NIK) and IKK1 (or IKKα), which phosphorylates p100. Subsequently, the C-terminal inhibitory domain of p100 is removed by proteasome resulting in the release of RelB:p52 NF-κB heterodimers into the nucleus. In comparison to the canonical RelA activity, the non-canonical pathway elicits a weak but sustained RelB activity, which induces genes involved in immune cell differentiation and immune organ development. In the absence of p100, RelB appears in the nucleus as a minor RelB:p50 NF-κB activity (16, 17). Notably, this constitutive RelB:p50 activity partially compensated for the absence of immune-organogenic RelB:p52 functions in *Nfkb2*^−/−^ mice (18).

Previous mechanistic analyses have identified molecular connections between the canonical and non-canonical NF-κB pathways. For example, canonical signaling induces the expression of genes encoding RelB and p100 from the respective NF-κB target promoters (15). A subpopulation of RelA binds to p100 and is activated by non-canonical signaling (16, 19–21). Conversely, IκBα retains a fraction of RelB and liberates a weak RelB NF-κB activity during canonical signaling in wild type MEFs (22, 23). More so, RelA and RelB heterodimers possess overlapping DNA binding and gene-expression specificities (23–26). Because NF-κB pathways are interlinked, we asked if constituents of the non-canonical pathway influence dynamical TNF signaling.

Here, we demonstrate that p100, a component of the immune-differentiating non-canonical pathway, is critical for the NF-κB system to discriminate between brief and chronic TNF signals. Brief TNF treatment, akin to chronic simulations, induced a biphasic NF-κB response in p100-deficient cells. However, the late NF-κB DNA binding activity induced in p100-deficient cells consisted of RelB:p50, which modified TNF-mediated gene controls in MEFs. Our study further revealed that RelA and RelB heterodimers bound to largely overlapping chromatin locations despite differences in the RelA-dependent and the RelB-mediated gene controls in p100-deficient cells. Mechanistically, NF-κB-driven RelB synthesis strengthened the basal RelB:p50 activity in p100-null cells upon TNF stimulation and produced this lasting NF-κB response. Finally, myeloma cells lacking p100 owing to genetic aberrations produced a long-lasting pro-survival RelB response to brief TNF stimulation. In sum, the NF-κB system engages distantly related molecular species with seemingly distinct biological functions for enforcing dynamical and gene controls of TNF signaling. Our work suggests that varied cellular abundance of p100 may also provide for a mechanism of tuning TNF responses in diverse physiological and pathological settings.

## Results

### A Mathematical Model of the Integrated NF-κB System Predicts a Role of p100 in TNF Signaling

**Figure 1:**
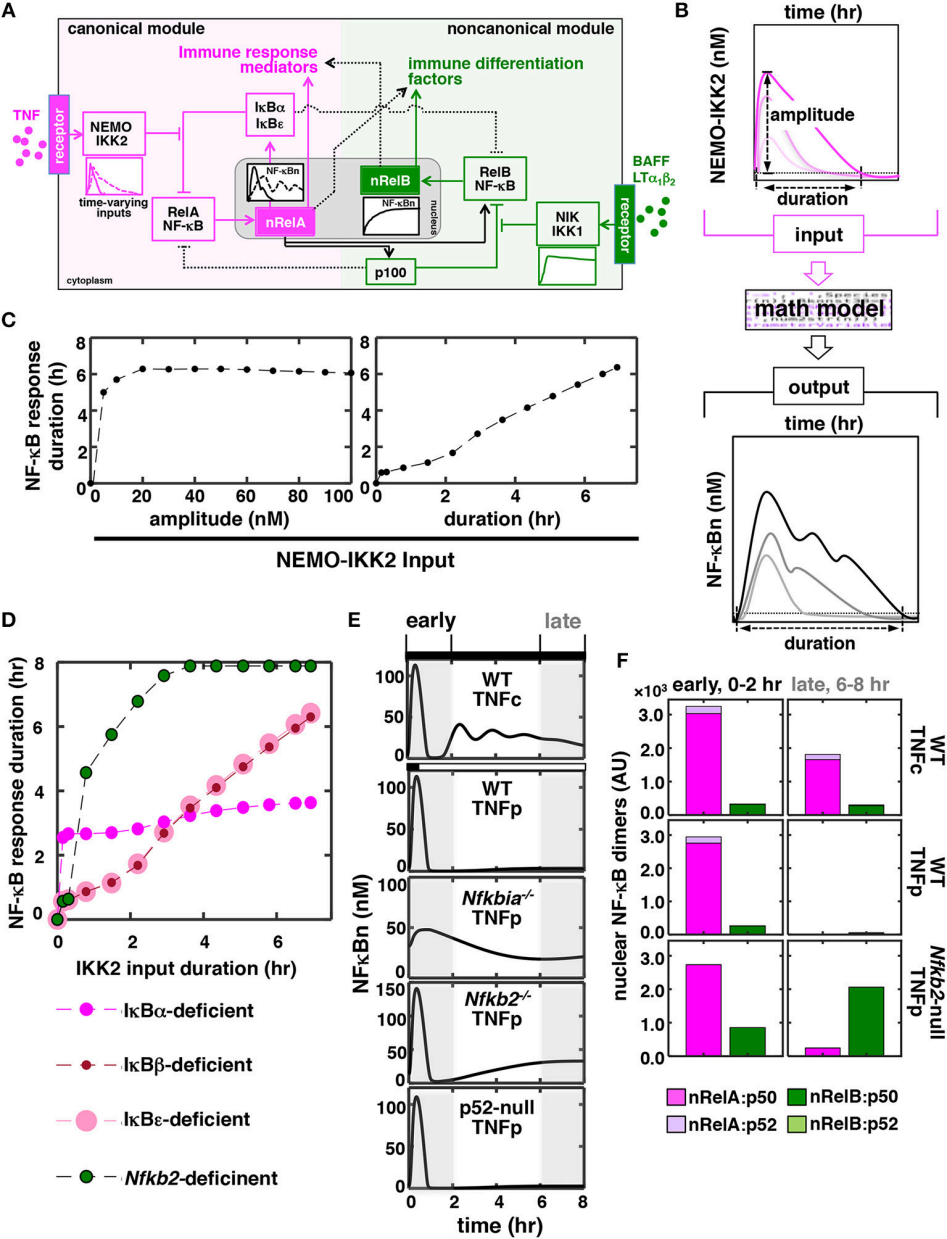
*In silico* studies identify a role of p100 in discriminating between time-varying TNF inputs. **(A)** A graphical depiction of the NF-κB system. TNF through the canonical pathway (magenta) dynamically regulates the activity of RelA:p50 heterodimers, which mediate the expression of immune response genes. BAFF or LTα_1_β_2_ induces a distinct RelB NF-κB activity via a separate non-canonical pathway (green) for driving the expression of immune differentiation factors. However, these two NF-κB pathways are molecularly connected and display certain overlap in relation to gene expressions. Solid and dotted black lines represent major cross-regulatory mechanisms and those involving less-preferred biochemical reactions, respectively. NF-κBn, nuclear NF-κB activity. nRelA and nRelB represent corresponding nuclear heterodimers. **(B)** Schema describing *in silico* production function analyses. Briefly, theoretical IKK2 activity profiles of various peak amplitudes and durations were fed into the mathematical model, and NF-κBn responses were simulated in a time-course. Durations were estimated as the time elapsed above a specific threshold value, which was determined as the sum of the basal NF-κB or IKK activity and 5% of the corresponding basal-corrected peak activity, in the corresponding activity curves. **(C,D)** Graph plot of the duration of simulated NF-κBn responses as a function of the peak amplitude or the duration of theoretical IKK2 inputs. IKK2 activities of various peak amplitude but with invariant 8 h of duration (**C**, left) or with various durations but identical 60 nM peak amplitude (**C**, right and **D**) were used. Computational simulations involved **(C)** the WT system and **(D)** the indicated mutant systems. **(E)** *In silico* studies revealing NF-κBn responses in a time-course in WT and various mutant systems. Experimentally derived IKK2 activity profiles, obtained using MEFs treated with TNF either chronically (TNFc) or for 0.5 h (TNFp), were used as model inputs. Early (0–2 h) and late (6–8 h) phases have been marked using gray boxes. **(F)** Computational modeling predicting TNFp-induced nuclear activities of RelA and RelB heterodimers in WT and *Nfkb2*-null systems. Early and late activities were determined as the area under the corresponding activity curve between 0 and 2 h and 6–8 h, respectively, subsequent to correction for basal values. AU, arbitrary unit.

Mathematical reconstructions of cellular networks offer insights on the underlying signal-processing mechanisms (27, 28). We developed a mathematical model (see Supplementary Materials for details), which depicted the NF-κB system consisting of interlinked canonical and non-canonical modules (Figure 1A), for probing dose-duration control of TNF signaling *in silico*. In this model, IκBs and inhibitory p100 complexes both regulated nuclear NF-κB (NF-κBn) activities. For varying dose and treatment duration, TNF activates IKK2 with diverse kinetic profiles. Accordingly, we used theoretical IKK2 activity profiles of varying peak amplitude or duration as model inputs (Figure 1B; Figures S1A,B). Our computational simulations broadly captured the previously described NF-κB dynamics (4, 6). For example, the duration of NF-κBn response was insensitive to changes in the amplitude of IKK2 signal but proportionately increased as a function of the duration of IKK2 input (Figure 1C). Simulating mutant cell systems devoid of one or the other NF-κB regulators, we examined their role in this dynamical control. Remarkably, our computational analyses suggested an aberrant NF-κB control in the *Nfkb2*-deficient system where even short-duration IKK2 inputs produced prolonged NF-κBn responses (Figure 1D).

To investigate further dynamical TNF signaling *in silico*, we fed experimental IKK2 activity profiles obtained using MEFs treated with TNF into our mathematical model as inputs (Figure S1C) (6, 20). Indeed, long-lasting IKK2 activity associated with chronic TNF treatment (TNFc) triggered a prolonged, biphasic NF-κBn response consisting of RelA:p50 heterodimers in our simulation studies (top panels, Figures 1E,F). Short-lived IKK2 input related to brief 0.5 h of TNF treatment (TNF pulse, TNFp) produced only a transient 1 h of NF-κBn response. As expected, a weakened negative feedback extended the TNFp-induced NF-κB response beyond 1 h in the IκBα-deficient system. Corroborating our studies involving theoretical IKK2 inputs, computational simulation of the TNFp regime in the *Nfkb2*-deficient system produced a prolonged NF-κBn response, whose temporal profile was somewhat comparable to that of the TNFc-induced NF-κBn activity (Figure 1E). The prolonged activity induced in the *Nfkb2*-deficent system was biphasic where the late phase lasted for more than 8 h. However, this late activity was absent in the p52-null system, where p100 was expressed but its conversion into p52 was not permitted. Because p100 deficiency triggers also canonical RelB:p50 activation, we probed the dimer composition of this late-acting NF-κB response. Our mathematical model included the description of four NF-κB heterodimers, namely RelA:p50, RelA:p52, RelB:p50, and RelB:p52. Recapitulating previously published experimental data, our simulation studies revealed that the TNF-induced NF-κBn activity consisted of mostly RelA:p50 in the WT system with only a minor amount of RelA:p52 and RelB:p50 heterodimers (Figure 1F). Our computational model further indicated that primarily signal-induced nuclear accumulation of RelB:p50 heterodimers generated the late-acting NF-κBn response to TNFp in the *Nfkb2*-deficient system (Figure 1F). Therefore, our mathematical modeling studies predicted a role of the non-canonical signal transducer p100 in producing appropriate NF-κBn responses to time-varying TNF inputs.

### p100 Restrains Late-Acting RelB:p50 NF-κB Response to Brief TNF Stimulation

**Figure 2:**
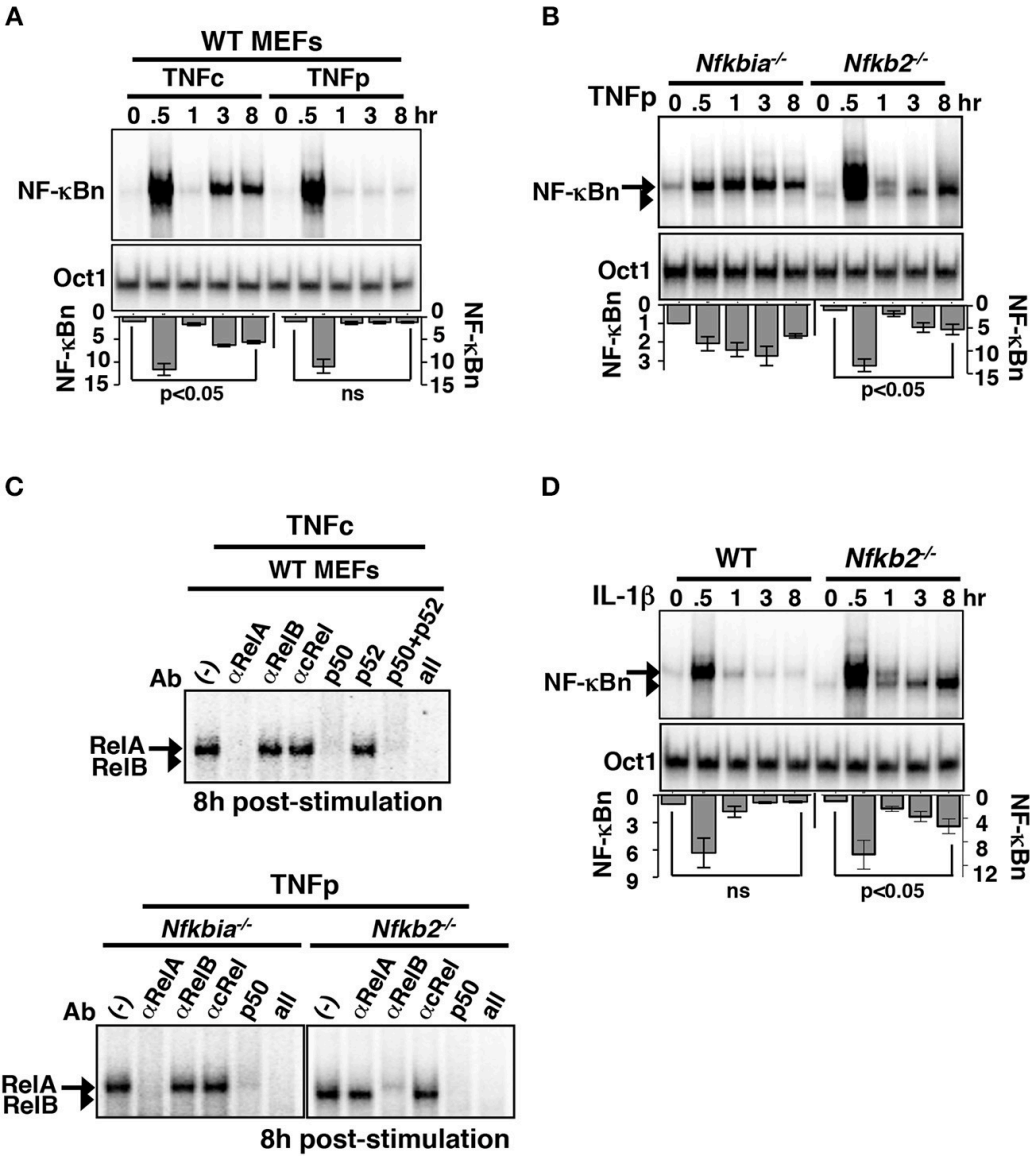
Brief TNF stimulation of *Nfkb2*^−/−^ MEFs induce an additional late NF-κB activity composed of RelB:p50 heterodimers. **(A)** WT MEFs were subjected to TNFc or TNFp treatments, cells were harvested at the indicated time-points after the commencement of stimulations, and NF-κBn DNA binding activities were resolved in EMSA (top panel). DNA binding activity of Oct1 served as a loading control (middle panel). Bottom: signals corresponding to NF-κBn were quantified from four independent experiments and presented in relation to the basal activity in a bargraph. Statistical significance was evaluated using Student's *t* test. **(B)** EMSA comparing NF-κBn induced in a time-course in *Nfkbia*^−/−^ and *Nfkb2*^−/−^ MEFs upon TNFp treatment (top panel). As determined in **(C)**, the arrow and the arrowhead indicate RelA-containing and RelB-containing NF-κB complexes, respectively. Bottom: quantitative analysis of the total NF-κBn activities from four experimental replicates. **(C)** Composition of the NF-κBn activities that persisted after 8 h of TNFp treatment in *Nfkbia*^−/−^ and *Nfkb2*^−/−^ MEFs, was determined in the shift-ablation assay. Antibodies against the indicated NF-κB subunits were used for ablating the respective DNA binding complexes in EMSA. Data represents two independent experiments. **(D)** Time-course analysis of NF-κB DNA binding activity induced upon IL-1β treatment of WT or *Nfkb2*^−/−^ MEFs (top panel). Bottom: quantified NF-κB signal intensities; data represent four experimental replicates. Quantified data presented in this figure are means ± SEM.

To verify experimentally the predictions of our mathematical model, we treated MEFs, immortalized using NIH 3T3 protocol, with TNF and measured the resultant NF-κBn activities in a time-course using the electrophoretic mobility shift assay (EMSA). TNFc treatment of WT cells induced a biphasic NF-κBn response comprising of an early peak, which lasted for ~1 h, and a gradually weakening second phase between 3 and 8 h (Figure 2A). TNFp treatment of WT MEFs produced the early peak activity, which was substantially broadened in TNFp-treated *Nfkbia*^−/−^ cells lacking IκBα (Figures 2A,B). TNFp indeed induced a prolonged NF-κBn response in *Nfkb2*^−/−^ MEFs that consisted of an early peak and a progressively strengthening second phase (Figure 2B). Of note, TNFc generated a similar biphasic activity in *Nfkb2*^−/−^ cells (Figure S2A). Our shift-ablation assay confirmed that the late NF-κBn DNA binding activity induced in WT cells in response to TNFc was composed of mostly RelA:p50 heterodimers (Figure 2C). Similarly, the late NF-κBn activity induced by TNFp in *Nfkbia*^−/−^ cells consisted of RelA:p50. It was earlier shown that p100 deficiency alters the RelB homeostasis, where a subpopulation of RelB translocate into the nucleus, and yet another fraction is sequestered by IκBα and activated upon canonical signaling (16, 17, 20, 22, 23). Our shift-ablation assay corroborated these studies. We noticed in *Nfkb2*^−/−^ MEFs a low level of basal RelB:p50 activity; targeting IκBα-bound complexes, TNFp further augmented this RelB activity at 0.5 h post-stimulation that was diminished to the basal level by 1 h (Figure S2B). In addition, brief TNF stimulation produced a robust late-acting RelB response, which persisted in the nucleus of *Nfkb2*^−/−^ MEFs even 16 h after stimulation (Figure 2C; Figure S2B). Furthermore, IL-1β, which induces NF-κB signaling transiently in WT cells (6), produced a similar late RelB:p50 activity in *Nfkb2*^−/−^ MEFs (Figure 2D; Figure S2C). Our studies suggested that p100 imparted dynamical NF-κB control by preventing late-acting RelB:p50 response to short-lived IKK2 signals generated by pro-inflammatory cytokines. However, deficiency of p100 and that of the well-articulated negative feedback regulator, IκBα caused distinct aberrations with respect to the temporal profile and the composition of the signal-induced nuclear NF-κB activity.

Of note, we relied on bulk measurements of transcription factors present in the nuclear extracts. Therefore, our study does not rule out that p100 deficiency triggers an asynchronous, oscillatory RelB:p50 response to TNFp at the single-cell level. Cellular heterogeneity may also amount to two distinct cell population with only one sustaining an elevated RelB:p50 activity—this may in fact lead to an underestimation of late RelB:p50 response in our bulk measurement based analyses.

### Dissecting Molecular Mechanism Underlying Late RelB:p50 Response to Brief TNF Stimulation in the Absence of p100

**Figure 3:**
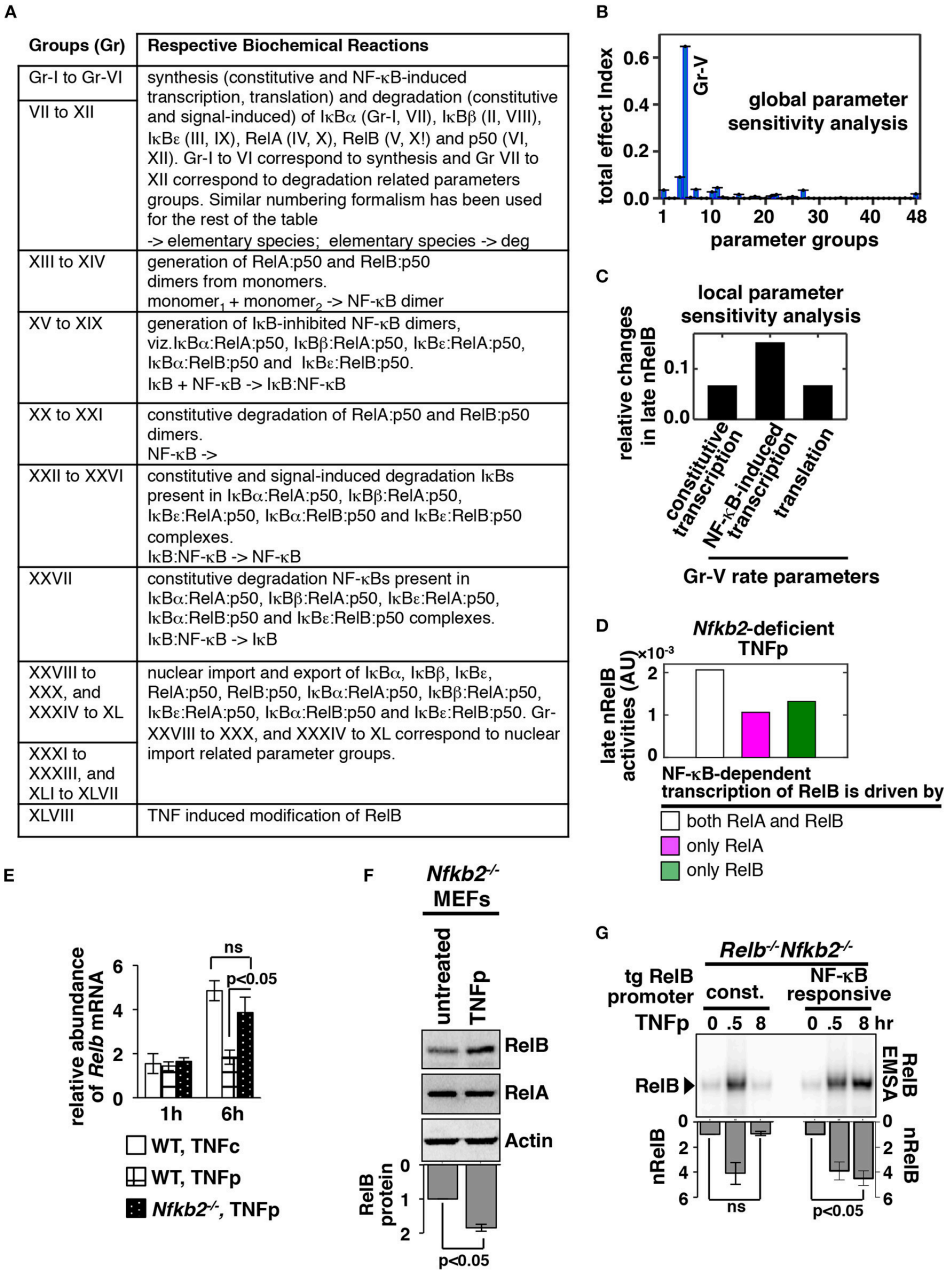
NF-κB-dependent RelB synthesis promotes the late RelB:p50 activity induced upon brief TNF treatment of *Nfkb2*^−/−^ cells. **(A)** Parameter groups analyzed in the variance-based, multiparametric sensitivity analysis. **(B)** Variance-based multiparametric analysis revealing the total effect index, which represent the effect of the parameter uncertainty on the late (6–8 h) RelB:p50 activity induced by TNFp in the *Nfkb2*-deficient system for the individual parameter groups. Standard bootstrapping was used for estimating error ranges. Gr, group. Gr-V consists of parameters related to RelB synthesis; including constitutive and NF-κB induced transcriptions as well as translation. **(C)** Local sensitivity analysis revealing the effect of 10% increase in the indicated parameters belonging to Gr-V on the late RelB:p50 response to TNFp in the *Nfkb2*-deficient system. Differences in the basal-corrected, RelB:p50 activity between the unperturbed system and perturbed systems were scored. **(D)** Computational simulations of the late nRelB response to TNFp involving *Nfkb2*-deficient systems, where the expression of RelB mRNA is mediated by either RelA as well as RelB heterodimers or by RelA alone or by exclusively RelB. **(E)** WT and *Nfkb2*^−/−^ MEFs were treated with TNFp before being subjected to qRT-PCR analysis of *Relb* mRNA abundances normalized to that of *Actb* mRNA. WT MEFs treated with TNFc were used as control. Bargraphs demonstrate the abundances of mRNAs in TNF-treated cells relative to those measured in the untreated cells. Data represent four biological replicates. **(F)** *Nfkb2*^−/−^ MEFs were treated with TNFp and harvested at 6 h post-stimulation before being analyzed by Western blotting. Actin served as a loading control. Bottom: densitometric analysis of the relative abundance of RelB protein in whole cell extracts; data represent five biological replicates. **(G)** TNFp-induced nRelB activity in *Relb*^−/−^*Nfkb2*^−/−^ MEFs stably expressing RelB from a retroviral transgene (tg) either constitutively (const.) or from an NF-κB responsive promoter. Ablating RelA DNA binding with an anti-RelA antibody, residual nRelB activities were revealed by RelB-EMSA. Data represent four independent experiments. Quantified data presented in this figure are means ± SEM.

Sensitivity analysis provides information on regulatory mechanisms governing the functioning of the modeled network (28). In local sensitivity analyses, rate parameters are individually altered; multiple parameters are changed simultaneously in multiparametric analyses. By estimating the effect of parameter perturbation on the model output, relative importance of the associated biochemical reaction in signal processing is determined. Utilizing a variance-based, multiparametric sensitivity analysis method (29), we investigated the biochemical mechanism underlying late-acting RelB:p50 response to TNFp in the *Nfkb2*-deficient system. We assembled the large number of model parameters into 48 distinct groups (Figure 3A). Each of these groups consisted of functionally related biochemical parameters associated with a specific molecular species (see Table S5 for a detailed description on parameter grouping). For instance, kinetic rate parameters associated with the synthesis of IκBα, including constitutive and NF-κB-responsive transcriptions as well as translation, were grouped together. Using Monte Carlo sampling, we explored the parameter space surrounding the nominal values simultaneously among the different parameter groups. The effect of parameter uncertainty for individual parameter groups on the late RelB:p50 activity was summarized as the total effect index (29). Group-V showed a substantially high total effect index indicating that parameters belonging to this group played a dominant role in determining the late RelB:p50 response (Figure 3B; Figure S3A). Group-V consisted of rate parameters associated with NF-κB-driven and constitutive syntheses of *Relb* mRNA as well as translation of *Relb* mRNA. In a local sensitivity analysis, we then distinguished between these Group-V parameters for their relative contributions in eliciting late RelB:p50 activity. We introduced a 10% increase in the individual rate parameters and the resultant effect on the late RelB:p50 response was scored subsequent to data normalization. Our analysis indicated that particularly NF-κB-mediated transcription of *Relb* promoted the late RelB:p50 response to TNFp in the *Nfkb2*-deficient system (Figure 3C). Both RelA and RelB heterodimers are capable of inducing the expression of *Relb* mRNA from the endogenous NF-κB target promoter (17, 23). Indeed, our computational model included the description of RelA, as well as RelB, mediated synthesis of RelB. To understand the contribution of these individual processes in late-acting RelB:p50 response, we abrogated either RelA-dependent or RelB-mediated RelB transcriptions in our model. Our computational simulations revealed that disruption of either RelA-mediated or RelB-driven transcription of RelB diminished the late RelB:p50 response in p100-deficient cells (Figure 3D). Therefore, our computational studies suggested that RelA-mediated transcription of *Relb* mRNA was not sufficient and autoregulatory synthesis of RelB was important for modifying dynamical TNF controls.

We tested these computational predictions experimentally. We observed that TNFc activated delayed expression of *Relb* mRNA, which is encoded by a NF-κB target gene, in WT MEFs (Figure 3E). Consistent with the lack of late NF-κBn activity in TNFp-treated WT MEFs, TNFp-induced expressions of *Relb* mRNA were less prominent in these cells (Figure 3E). However, TNFp treatment of *Nfkb2*^−/−^ MEFs led to heightened synthesis of *Relb* mRNA and protein at 6 h post-stimulation that temporally coincided with the late RelB activity observed in these cells (Figures 3E,F). Using retroviral constructs, we then expressed RelB from either a constitutive or a NF-κB responsive transgenic promoter in *Relb*^−/−^*Nfkb2*^−/−^ MEFs. TNFp treatment induced the accumulation of RelB mRNA in *Relb*^−/−^*Nfkb2*^−/−^ cells expressing RelB from the NF-κB-driven, but not constitutive, promoter (Figure S3B). Furthermore, TNFp triggered the late RelB:p50 activity only in engineered cells expressing RelB from the NF-κB responsive promoter, but not in cells expressing RelB from the constitutive promoter (Figure 3G). These results suggested that NF-κB-induced synthesis of RelB was required for triggering the late-acting RelB:p50 response to TNFp in the absence of p100. Therefore, our combined mathematical and biochemical analyses established that NF-κB-driven sustained RelB production promoted progressive nuclear accumulation of RelB:p50 heterodimers in response to brief TNF stimulation of p100-deficient cells.

### RelB:p50 Modify the TNF-Activated Gene-Expression Program in *Nfkb2^−/−^* MEFs

**Figure 4:**
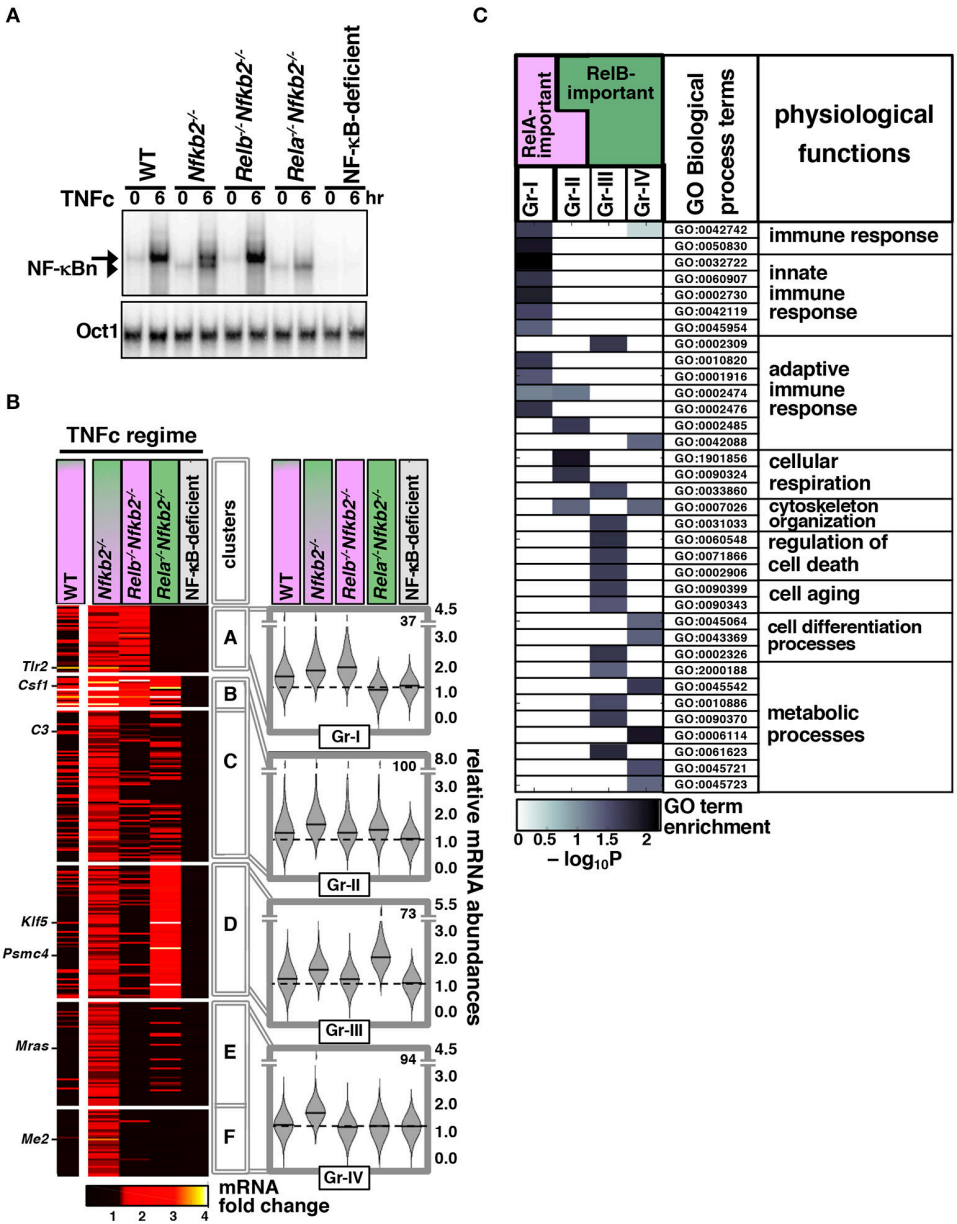
Global analyses reveal distinct gene controls by the TNF-activated RelB:p50 heterodimer. **(A)** MEFs of the indicated genotypes were treated with TNFc for 6 h before being subjected to EMSA. The data represents three independent experiments. **(B)** In our microarray mRNA analysis, we considered genes with high confidence detection in biological replicates across various knockout MEFs, and at least 1.3 fold increase in the average expression upon 6 h of TNFc treatment in *Nfkb2*^−/−^ cells, but <1.3 fold average induction in NF-κB-deficient cells to arrive onto a list of 304 genes. Heatmap demonstrates TNF-induced fold changes in the expressions of these genes in the indicated knockout cells clustered using the partition around medoids algorithm. A representative data using WT MEFs has been indicated in the left column. The resultant six gene-clusters were arranged into four gene-groups. Representative genes belonging to different groups have been indicated. Right: violin plots show relative frequency distributions of fold change values and corresponding medians for various genotypes as well as the number of members in each gene-group. **(C)** Functional enrichment of various Gene Ontology for Biological Process terms in the indicated gene-groups was determined by topGO. A subset of highly enriched terms in either of the gene-groups is highlighted. Broad physiological functions associated with these GO terms have been also indicated.

Next, we sought to determine the gene-expression specificity of RelB:p50 in microarray mRNA analysis (Materials and Methods, Supplementary Materials). For side by side comparison of gene controls by RelA:p50 and RelB:p50, we focused on TNFc regime, which produced equivalent nuclear activity of these two heterodimers at 6 h post-stimulation in *Nfkb2*^−/−^ MEFs (Figure 4A) (23). To dissect genetically heterodimer-specific gene expressions, we additionally examined *Relb*^−/−^*Nfkb2*^−/−^ MEFs, which activated exclusively RelA:p50 upon TNFc treatment, and *Rela*^−/−^*Nfkb2*^−/−^ cells, which elicited solely RelB:p50 response (Figure 4A). As controls, we used WT MEFs, which induced RelA:p50 activity in response to TNFc, and NF-κB-deficient cells, which lacked all three transcription-competent NF-κB subunits RelA, RelB, and cRel. In our microarray mRNA analysis, we first considered genes whose expression was induced at least 1.3 fold at 6 h post-TNFc treatment in *Nfkb2*^−/−^ MEFs, but not in NF-κB-deficient cells. Accordingly, we arrived at a list of 304 NF-κB dependent genes. Based on their differential expressions in *Relb*^−/−^ *Nfkb2*^−/−^ and *Rela*^−/−^*Nfkb2*^−/−^ MEFs, we cataloged these NF-κB-dependent genes into six distinct clusters, which were arranged further into four gene-groups (Gr-I to Gr-IV; Figure 4B; Supplementary Materials, Materials and Methods). Gr-I genes were induced in WT, *Nfkb2*^−/−^ and *Relb*^−/−^*Nfkb2*^−/−^ MEFs that possessed the RelA:p50 activity (Figures 4A,B). Gr-II genes were activated either in the presence of RelA:p50 in WT, *Nfkb2*^−/−^ and *Relb*^−/−^ *Nfkb2*^−/−^ MEFs or in RelB:p50-containing *Rela*^−/−^ *Nfkb2*^−/−^ cells. Genes belonging to Gr-III required RelB:p50 for their expressions; they were induced in *Nfkb2*^−/−^ or *Rela*^−/−^*Nfkb2*^−/−^ MEFs, but not in WT or *Relb*^−/−^ *Nfkb2*^−/−^ cells. Gr-IV genes were activated only in *Nfkb2*^−/−^ MEF possessing both RelA:p50 and RelB:p50 activities. Our analyses of knockout cells suggested that RelB:p50 heterodimer could mediate the expression of a subset (Gr-II) of NF-κB-target genes activated by TNF in WT cells involving RelA:p50. Of note, previous studies also reported that RelA:p50 and RelB:p50 function redundantly in mediating the expression of certain pro-inflammatory genes, such as those encoding RANTES, as well as pro-survival genes, such as those encoding cFLIP (20, 23). Intriguingly, RelB:p50, either alone (Gr-III) or in collaboration with RelA:p50 (Gr-IV), activated additional genes in *Nfkb2*^−/−^ MEFs that were not normally induced in WT MEFs. Therefore, p100 modified transcriptional responses to TNF in MEFs involving both RelA- as well as RelB-dependent mechanisms.

We then subjected these gene-groups to gene ontology (GO) analyses. Consistent with the well-articulated role of the canonical pathway in immune-activating TNF signaling, Gr-I and Gr-II comprising TNFc-induced RelA-important genes were enriched for GO terms associated with innate and adaptive immune responses (Figure 4C). Gr-II also scored highly for terms linked to cellular respiration. Gr-III and Gr-IV consisting of RelB-important genes activated in p100-deficient cells were instead enriched for terms associated with cellular differentiation, aging, and cell death as well as metabolic processes. These RelB-important genes scored poorly for immune response related GO terms.

**Figure 5:**
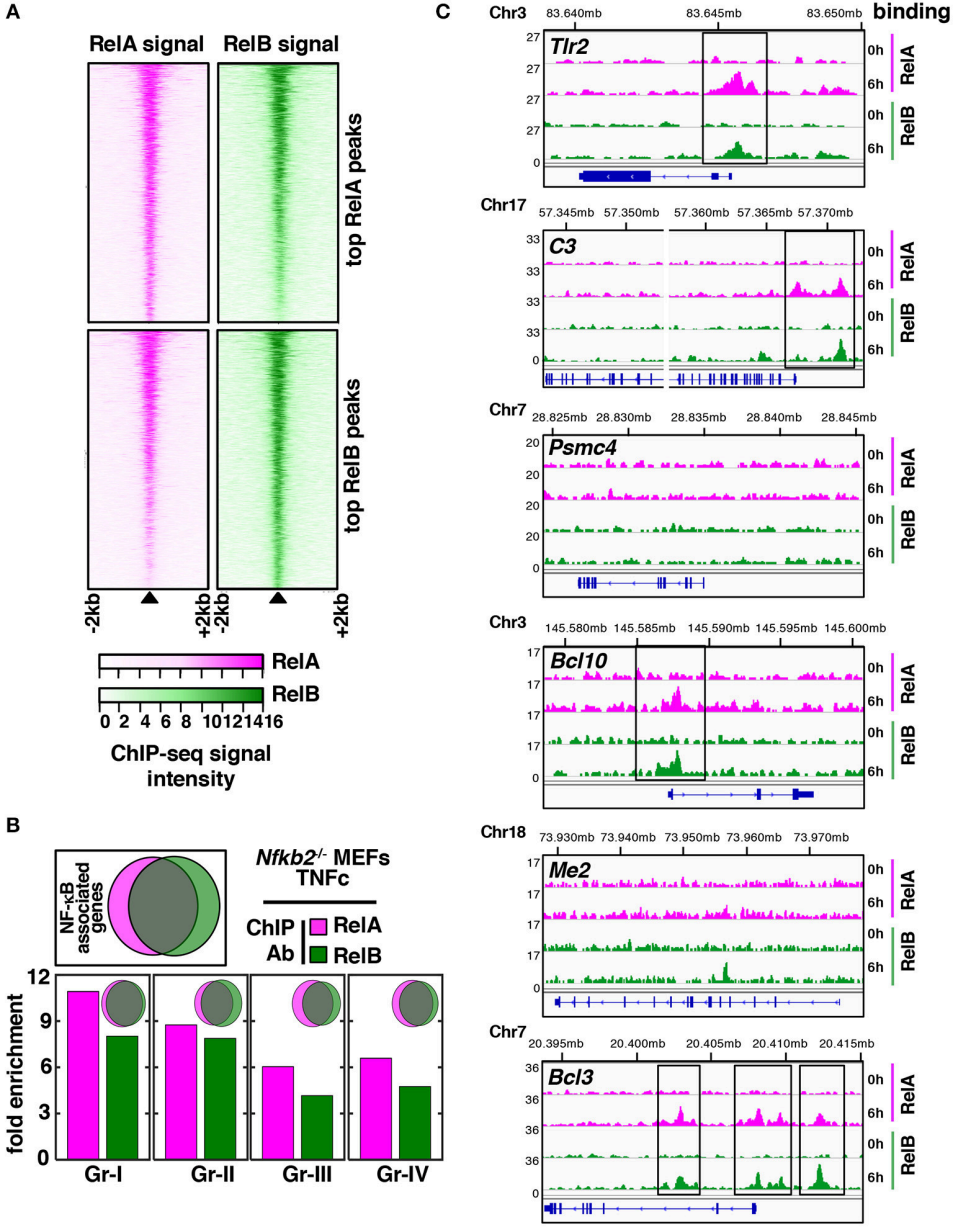
Overlapping genome-wide distributions of RelA and RelB in TNF-stimulated *Nfkb2*^−/−^ MEFs. **(A)** Using ChIP-seq analyses, we captured the genome-wide distribution of RelA and RelB in *Nfkb2*^−/−^ MEFs subjected to TNFc treatment for 6 h. A peak centered heatmap revealing RelA and RelB binding to chromatin locations surrounding top RelA or top RelB binding peaks. **(B)** Venn diagrams reveal overlap between RelA- (magenta) and RelB-associated (green) genes globally (top, left) or for the individual gene groups. Bar graphs reveal relative enrichment of RelA-associated and RelB-associated genes for various gene groups. **(C)** Representative browser tracks of RelA and RelB binding to genes belonging to various gene groups. The y-axis represents normalized reads per 10 million. Chromosomal locations of each gene in mm9 are shown above ChIP-seq tracks.

We asked if overlapping and distinct gene controls by RelA and RelB heterodimers were mediated at the level of chromatin binding. To address this, we subjected *Nfkb2*^−/−^ MEFs to TNFc treatment for 6 h and subsequently performed chromatin immunoprecipitation using anti-RelA or anti-RelB antibodies followed by deep-sequencing (ChIP-seq) analysis (see Materials and Methods, Supplementary Materials). We then assessed the RelA as well as the RelB ChIPed-tag density around (± 2 kb) the center of the top 2077 RelA binding peaks (top panels, Figure 5A). Similarly, RelA and RelB binding surrounding the top 2241 RelB binding peaks were charted (bottom panels, Figure 5A). These top-ranking peaks were selected basing on their intensity as well as their rank in the irreproducible discovery rate test (30). Our peak-centered heatmap revealed that RelA and RelB bound to mostly overlapping chromatin sites and with almost similar proficiency. Next, we focused our analyses on Gr-I, Gr-II, Gr-III, and Gr-IV genes, which showed distinct requirements of NF-κB subunits for their expressions. We considered chromatin locations up to 50 kb from the transcription start site for assigning peaks to a given gene. Our analyses revealed that RelA or RelB recruitments to chromatin sites in TNFc-stimulated *Nfkb2*^−/−^ cells were equivalently enriched for all four gene-groups (see bargraphs Figure 5B). We indeed noticed a substantial overlap between RelA- and RelB- associated genes globally and in the individual gene-groups (see Venn diagrams, Figure 5B). Finally, we examined browser tracks of a select set of genes belonging to these gene-groups (Figure 5C). *Tlr2* belonging to Gr-I and *C3* belonging to Gr-II were bound by both RelA and RelB in TNFc-treated *Nfkb2*^−/−^ MEFs. Among the Gr-III genes, *Psmc4* did not recruit these NF-κB subunits but *Bcl10* engaged both RelA and RelB. Similarly, either RelA or RelB was not recruited to *Me2* belonging to Gr-IV, but both bound *Bcl3*. Therefore, despite genetic analyses revealing distinct sets of RelA- and RelB-important genes, our ChiP-seq analyses suggested that RelA and RelB bound to largely overlapping chromatin sites, and that a subset of RelB-important genes circumvented NF-κB binding for their expressions.

Taken together, our genome-scale analyses implied that RelB:p50 were capable of modifying the TNF-induced gene-expression program in MEFs. RelB:p50 activated by TNFc in *Nfkb2*^−/−^ cells induced a distinct set of genes, which were not induced by RelA:p50 in WT cells and encoded functions unrelated to immune processes. However, this distinct gene control was not attributed to specific chromatin binding by RelB:p50 heterodimers.

### p100 Determines Specificity and Dynamical Control of TNF-Mediated Gene Expressions

**Figure 6:**
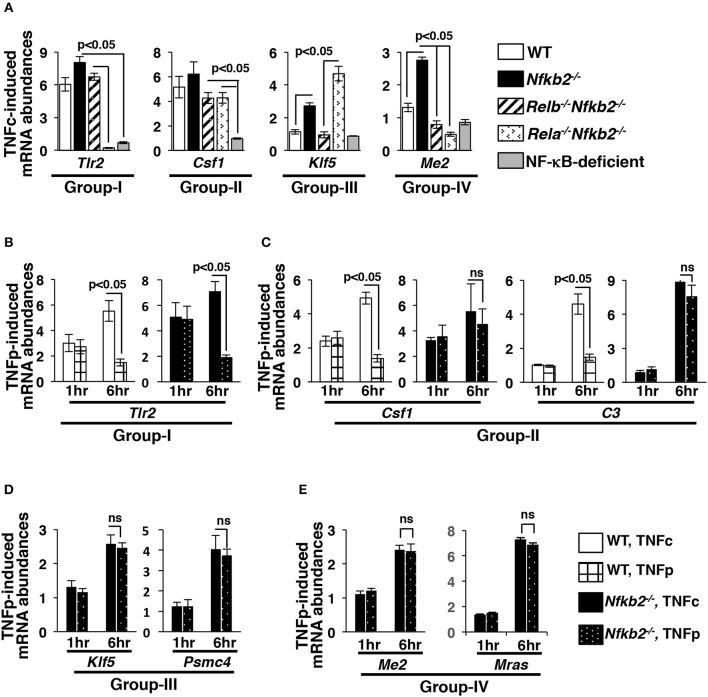
Brief TNF stimulation induces delayed, RelB-dependent gene expression in *Nfkb2*^−/−^ MEFs. **(A)** WT and knockout MEFs were subjected to TNFc treatment for 6 h, and the expressions of the indicated genes belonging to different gene-groups were measured by qRT-PCR. Bargraphs demonstrate the abundances of the corresponding mRNAs in stimulated cells relative to those measured in untreated cells. Data represent three biological replicates. **(B–E)** WT and *Nfkb2*^−/−^ MEFs were subjected to TNFc or briefly stimulated with TNF for 0.5 h (TNFp), and subsequently cells were harvested at the indicated time-points before being subjected to qRT-PCR analyses. Bargraphs demonstrate TNF-induced expressions of the indicated genes, representing various gene-groups, in relation to untreated cells. Data represent four independent experiments. Quantified data presented in this figure are means ± SEM.

We further validated our microarray data for a select set of genes representing different gene-groups using quantitative real time-polymerase chain reaction (qRT-PCR) analyses. TNFc treatment for 6 h triggered the RelA-dependent expression of Gr-I gene *Tlr2* in WT, *Nfkb2*^−/−^ as well as *Relb*^−/−^*Nfkb2*^−/−^ MEFs and not in *Rela*^−/−^*Nfkb2*^−/−^ cells (Figure 6A). TNFc induced *Csf1* belonging to Gr-II, whose members were activated redundantly by RelA:p50 and RelB:p50 in our microarray studies, not only in WT and *Nfkb2*^−/−^ MEFs but also in *Relb*^−/−^*Nfkb2*^−/−^ and *Rela*^−/−^*Nfkb2*^−/−^ cells. As expected, RelB was both necessary and sufficient, and mediated the expression of Gr-III gene *Klf5* in *Nfkb2*^−/−^ and *Rela*^−/−^*Nfkb2*^−/−^ MEFs but not in WT and *Relb*^−/−^*Nfkb2*^−/−^ cells. Consistent to the proposed requirement of both RelA:p50 and RelB:p50 for the expression of Gr-IV genes, *Me2* and *Mras* was induced selectively in *Nfkb2*^−/−^ MEFs. NF-κB-deficient cells did not activate these genes in response to TNF. Therefore, our qRT-PCR analyses substantiated our genome-scale data highlighting distinct gene controls by RelA and RelB heterodimers.

Sustained expression of NF-κB-dependent genes require prolonged RelA:p50 nuclear activity, such as those produced in WT cells by TNFc (4, 8, 31, 32). On other hand, it has been found that transient RelA:p50 activity elicited by TNFp is inadequate for the continued expression of NF-κB-target genes. Because TNFp stimulated a prolonged nuclear activity of RelB:p50 in *Nfkb2*^−/−^ MEFs, we asked if TNFp triggered persistent expression of NF-κB-dependent genes in p100-deficient cells. Our time-course analyses demonstrated that TNFc induced progressive accumulation of mRNAs encoding Gr-I and Gr-II genes in WT as well as *Nfkb2*^−/−^ MEFs between 1 and 6 h post-treatment (Figures 6B,C). As expected, TNFp failed to sustain the expression of these RelA-important genes in WT cells. *Nfkb2*^−/−^ MEFs upheld the expression of Gr-II genes, which could be activated by either RelA or RelB factors, in response to TNFp at 6 h post-stimulation. Akin to TNFc, TNFp additionally stimulated delayed expressions of RelB-important genes, which included Gr-III as well as Gr-IV genes, in *Nfkb2*^−/−^ MEFs at 6 h post-stimulation (Figures 6D,E). These genes were not activated in WT MEFs even upon TNFc stimulation. Collectively, p100 enforced both dynamical control and the specificity of the TNF-induced gene-expression program. Brief TNF stimulation of p100-deficient cells triggered a prolonged RelB:p50 activity, which not only sustained the expression of a subset of RelA-important genes but also induced delayed expressions of metabolic and immune-differentiation related genes, which were not normally activated by RelA:p50 in WT MEFs.

### Repeated Pulses of TNF Strengthen Late-Acting RelB:p50 Response in *Nfkb2^−/−^* Cells

**Figure 7:**
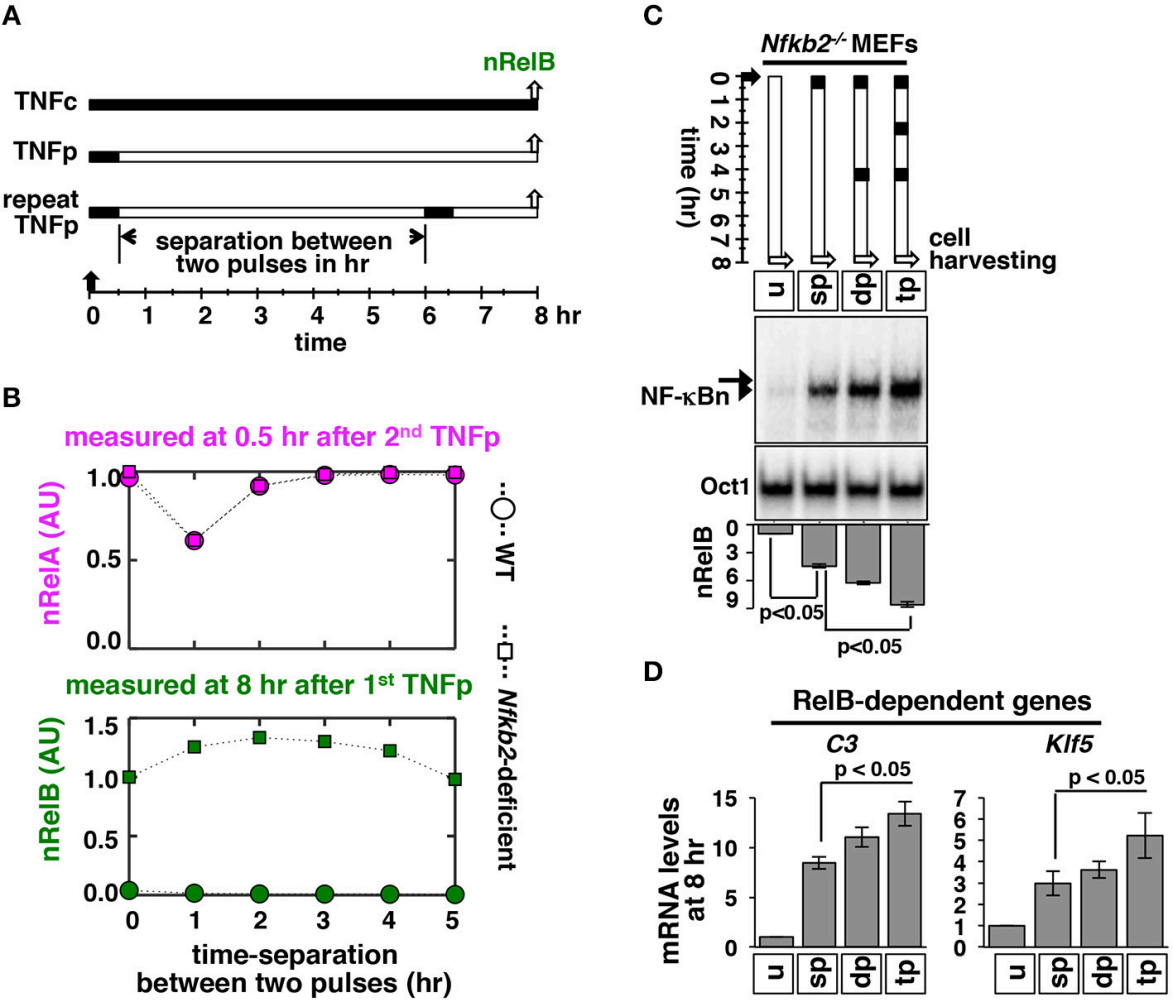
Repeated TNF pulses of *Nfkb2*^−/-^ cells strengthen late RelB:p50 signaling. **(A)** Schema describing repeated TNFp regime: the short-lived IKK2 input associated with the TNFp regime was fed into the model successively with varied separation time between two TNF pulses and corresponding NF-κBn was simulated. **(B)** Computational studies revealing the early nRelA activity induced 0.5 h after the second TNFp (top) and the late nRelB activity induced 8 h after the first TNFp (bottom) as a function of the separation time between two successive pulses in WT and *Nfkb2*-deficient systems. The early and late activities were normalized to those induced in response to a single pulse in the *Nfkb2*-deficient system. **(C)** *Nfkb2*^−/−^ MEFs were treated with either a single TNFp (single pulse, sp) or two successive TNFp separated by 4 h (double pulse, dp) or three pulses at 2 h intervals (triple pulse, tp). Cells were harvested 8 h after the first pulse and analyzed for NF-κBn by EMSA. *u* denotes untreated. Bottom: quantitative analysis of the nRelB activities; data represent three experimental replicates. **(D)** *Nfkb2*^−/−^ MEFs were subjected to the indicated treatments; cells were harvested 8 h after the first pulse and expressions of the indicated genes were measured by qRT-PCR. Bargraphs represent three biological replicates. Quantified data presented in this figure are means ± SEM of three biological replicates.

Within tissue microenvironment, macrophages secrete TNF in repeated bursts. Accordingly, effect of periodic TNF pulses on the nuclear NF-κB activity has been investigated *ex vivo*. When administered at short intervals, repeated TNF pulses produce a refractory state in WT cells leading to a diminishing RelA:p50 response (12, 33). Because p100 deficiency provoked an additional RelB:p50 response to brief TNF stimulation, we set out to examine mathematically as well as experimentally NF-κB activation in response to periodic TNF pulses in *Nfkb2*^−/−^ MEFs. Corroborating earlier studies, our computational simulations suggested that two consecutive TNF pulses separated by 1 h would lead to a weakened RelA:p50 response to the succeeding TNF pulse in both WT and *Nfkb2*-deficient systems (Figures 7A,B). Interestingly, our simulation studies also predicted that for a pulse separation of 1–4 h, a succeeding TNF pulse would augment the late RelB:p50 activity induced at 8 h by the preceding TNF pulse in the *Nfkb2*^−/−^deficient system. In our computational model, this heightened late RelB activity was accompanied by an increased abundance of *Relb* mRNA and protein (Figure S4A). Our experimental analyses substantiated that as compared to a single pulse, two or three consecutive TNF pulses augmented the late RelB activity as well as the abundance of *Relb* mRNA and protein in *Nfkb2*^−/−^ MEFs (Figure 7C; Figure S4B). Finally, double or triple TNF pulses enhanced the delayed expression of RelB-important genes in *Nfkb2*^−/−^ cells (Figure 7D). These studies identified an important role of p100 in the pulsatile TNF regime; although p100 did not participate in attenuating the RelA activity, it prevented escalating RelB:p50 response to periodic TNF pulses.

### Brief TNF Stimulation Triggers a Late-Acting, Pro-Survival NF-κB Response in p100-Deficient Myeloma Cells

**Figure 8:**
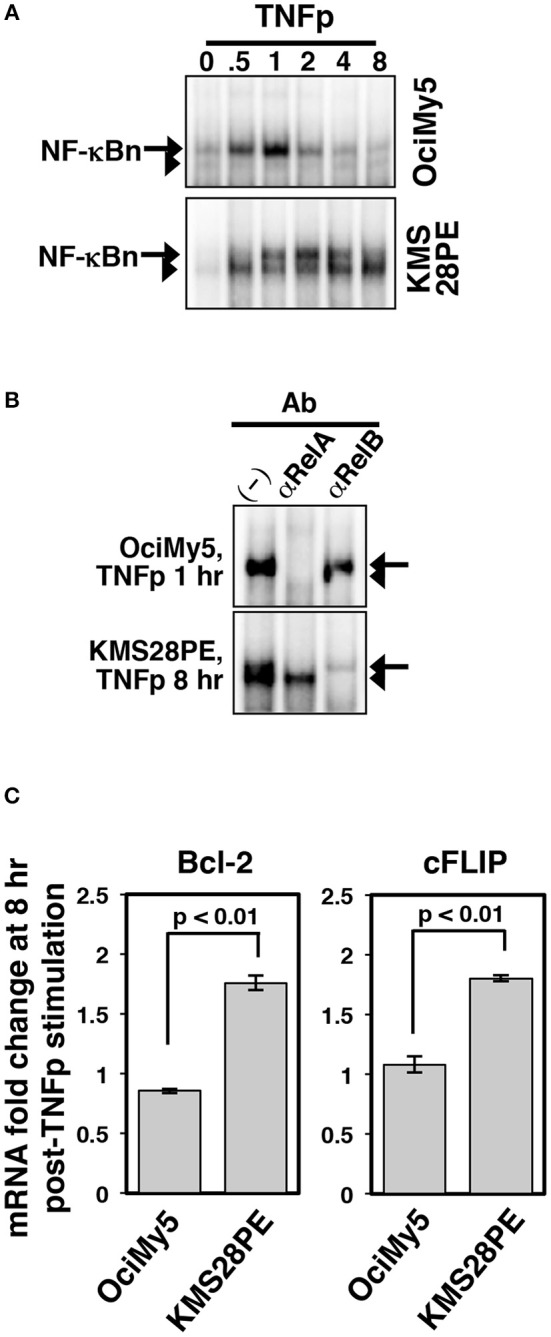
Altered dynamical NF-κB control in p100-deficient myeloma cells. **(A)** EMSA demonstrating NF-κBn activation upon brief TNF stimulation of the indicated human myeloma cell-lines. **(B)** The composition of the NF-κBn activities induced at 8 h post-TNFp stimulation in these myeloma cell-lines was determined by shift-ablation assay. **(C)** qRT-PCR revealing the expression of the indicated pro-survival genes in myeloma cells at 8 h post-TNFp treatment. The data represent means ± SEM of three biological replicates.

The non-canonical NF-κB pathway often accumulates gain-of-function mutations in multiple myeloma and these genetic aberrations were shown to completely degrade p100 in myeloma cells (34). It has been also suggested that TNF, which has a very short serum half-life, promotes survival of myeloma cells within the tumor microenvironment. We have earlier demonstrated that KMS28PE human myeloma cell-line was devoid of p100 because of non-canonical pathway mutations (23). Furthermore, chronic TNF treatment of these p100-depleted myeloma cells induced RelA:p50 as well as RelB:p50 complexes, both of which activated the expression of pro-survival factors. We asked if p100 deficiency modified the NF-κB response of myeloma cells to short-lived cytokine signals, such as those generated by TNFp. To this end, we compared KMS28PE cells with control OciMy5 cells, which preserved p100 expressions, in our biochemical studies. Brief TNF treatment induced a transient RelA NF-κB activity in OciMy5 cells that lasted about an hour (Figures 8A,B). In KMS28PE cells, TNFp induced a similar transient RelA activity that was mostly attenuated at 8 h post-stimulation. These p100-depleted myeloma cells also possessed constitutive RelB activity. Indeed, TNFp further induced progressive nuclear accumulation of RelB in KMS28PE that produced a strong RelB NF-κB DNA binding activity at 8 h post-TNFp treatment. Finally, our gene-expression studies revealed that TNFp induced late-expressions of mRNAs encoding pro-survival factors Bcl2 and cFLIP in KMS28PE cells (Figure 8C); these gene activities temporally coincided with the robust late-acting RelB response observed in these cells. TNFp stimulation did not induce pro-survival gene expressions in OciMy 5 cells. Our studies suggested that short-lived cytokine signals triggered a late-acting, pro-survival NF-κB response in human malignancies in the absence of p100.

## Discussion

**Figure 9:**
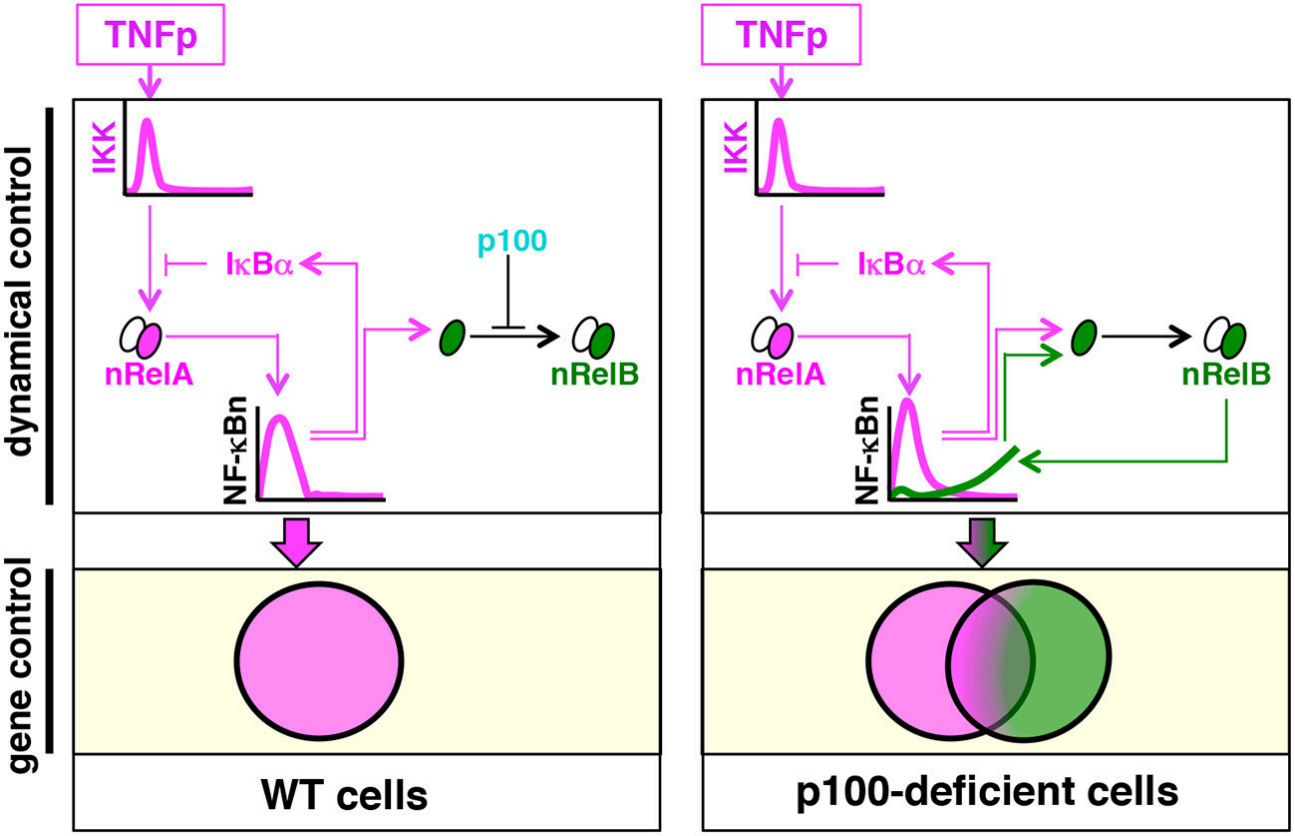
The proposed model explaining the role of p100 in the dynamical control and the gene expression of specificity of TNF-induced NF-κB signaling. Brief TNF stimulation elicits transient NF-κB activity composed of RelA:p50 heterodimers, which mediate the expression of immune response genes. An absence of p100 triggers a late RelB:p50 NF-κB activity in response to TNF that induces also the expressions of genes involved in metabolic and cellular differentiation processes.

Our study suggested that by insulating RelB heterodimers from the canonical NF-κB pathway, p100 enforced dynamical and gene controls of TNF signaling (Figure 9). As such, TNF engages the canonical pathway for activating RelA:p50 heterodimers, which induce the expression of specific immune response genes (2). Brief and chronic TNF treatments induce transient and long-lasting RelA:p50 activities, respectively, and produce distinct transcriptional responses. It is thought that the IκBα-mediated negative feedback hardwired in the canonical module enables the NF-κB system to distinguish between time-varied TNF inputs. p100 is rather known for transducing non-canonical NF-κB signals, which mediate nuclear activation of RelB heterodimers during immune differentiation (15). We found that the absence of p100 provoked a prolonged, biphasic NF-κB response to brief TNF stimulation. However, this late-phase NF-κB activity was composed of RelB:p50, and not RelA:p50, heterodimers. In *Nfkb2*^−/−^ cells subjected to brief TNF stimulation, RelB:p50 sustained the expression of a subset of immune response genes and also activated additional RelB-important genes, which encoded immune differentiation and metabolic functions. In response to periodic TNF pulses, the NF-κB system produces a refractory state that exerts a detrimental effect on the signal-induced RelA response and prevents unchecked RelA:p50 activity. In contrast to its inhibitory effect on the signal-induced RelA response, repeated TNF pulses strengthened the late-phase RelB:p50 activity in p100-deficient cells and augmented the expression of RelB-important genes.

TNF and other canonical pathway inducers do not cause degradation of p100, which is proteolyzed during non-canonical signaling. However, the TNF-activated canonical pathway induces the expression of *Nfkb2* mRNA, and the non-canonical signal transducer p100 interacts with RelA (19, 21). Indeed, a plausible role of p100 in TNF signaling was investigated earlier (20). Shih et al. (20) observed that p100 functions rather redundantly with IκBα in mediating post-induction attenuation of the RelA:p50 activity induced by chronic TNF treatment. Their study instead identified an important role of the p100-mediated negative feedback in regulating LPS-induced canonical RelA activity (20). In the absence of p100, however, a subpopulation of RelB is sequestered in the cytoplasm by IκBα, while the remainder translocates into the nucleus and produces a minor RelB:p50 NF-κB activity (17, 22, 23). It was shown that chronic TNF treatment, which degrades IκBα, strengthens this constitute RelB:p50 activity present in *Nfkb2*^−/−^ MEFs (16, 20, 23). The RelB:p50 activity induced in p100-deficient cells by TNFc paralleled the signal-induced RelA:p50 activity; it consisted of an early 0.5 h peak followed by an attenuated activity at 1 h and a late-acting response prevailing between 3 and 8 h (23). Our brief TNF stimulation regime instead generated contrasting temporal profiles of these two NF-κB heterodimers in *Nfkb2*^−/−^ cells; it induced a transient RelA:p50 activity but a prolonged RelB:p50 response (Figure 9). Our mechanistic studies suggested that NF-κB-driven RelB synthesis augmented the constitutive RelB:p50 activity present in *Nfkb2*^−/−^ MEFs in response to TNFp stimulation. In the absence of sequestration by p100, newly-synthesized RelB produced by TNFp translocated into the nucleus as RelB:p50 heterodimers, which generated enduring NF-κB response to short-lived TNF signal in *Nfkb2*^−/−^ MEFs.

Despite the established role of RelB in immune organogenesis, gene regulation by RelB heterodimers remain poorly understood. *In vitro* DNA interaction studies and ChIP-seq analyses showed that RelB and RelA heterodimers in fact bind to largely similar κB sequences (24–26). More so, genome-scale investigation indicated significant overlap between RelA and RelB with respect to the gene-expression specificity (22, 23, 25, 26). Our own global analyses involving *Nfkb2*^−/−^ MEFs revealed both overlapping and distinct gene functions of RelA:p50 and RelB:p50. We identified a subset of TNF-activated genes, whose expressions were induced redundantly by these two heterodimers. Indeed, expressions of these NF-κB-dependent genes were sustained by RelB:p50 in TNFp-stimulated *Nfkb2*^−/−^ MEFs. In addition, we characterized a distinct set of RelB-important genes, which were not normally activated by TNF and required RelB:p50 for their expressions. Surprisingly, our ChIP-seq analyses demonstrated equivalent binding of RelA and RelB heterodimers to the chromatin loci associated with RelB-important genes or genes that were activated redundantly by RelA or RelB. Our study, which involved the well-orchestrated MEF-based cell system subjected to a uniform cell-stimulation regime, indicated that DNA-protein interactions played a rather insignificant role in determining the gene-expression specificity of NF-κB heterodimers. In line with an earlier proposal (35), we speculate that the gene-expression specificity is largely contingent upon the interaction of NF-κB heterodimers with other transcription factors. Because certain RelB-important genes did not show NF-κB binding at their promoters, we do not rule out possible engagement of the RelB-driven transcriptional feedforward loop in mediating the expression of a subset of RelB-important genes (36). Future studies ought to elaborate the regulatory mechanism driving expressions of these RelB-important genes in immune cells.

In addition to modulating immune response, the pleiotropic cytokine TNF also contributes to immune differentiation, for example osteoclastogenesis (37). Interestingly, genetic studies often implicated non-canonical signal transducers RelB and p100 in TNF-dependent biological processes, including osteoclastogenesis (38–41). More so, it was reported that the abundance of p100 is subject to cell-type specific regulations with immature dendritic cells possessing only a minor amount (22). We propose that varied cellular abundance of p100 may provide for a mechanism of tuning TNF responses involving RelB:p50 in diverse physiological settings. Moreover, *Nfkb2* was shown to be frequently mutated in cancers and aberrant TNF signaling has been implicated in neoplastic diseases (42, 43). In particular, we have previously demonstrated that non-canonical pathway mutations completely degrade p100 in a subset of myeloma cell-lines. Our current study indicated that p100 depletion might enable RelB-dependent, late-acting expressions of pro-survival genes in myeloma cells subjected to brief TNF stimulation. Interestingly, a synthesis-dependent IRF4 activity was shown to protect myeloma cells in human patients (44). Furthermore, recent studies suggested that altered metabolism exacerbates malignant growth in human cancers (43). In this context, it will be important to determine if the synthesis-driven RelB activity, which was augmented upon periodic TNF pulses, caused abnormal metabolism in human malignancies with dysfunctional p100. In other words, our mechanistic studies, which involved MEF-based model cell culture system, should be further extended to analyze immune cells and disease-associated cells for unraveling physiological and patho-physiological significance of p100-mediated control of TNF signaling. Interestingly, previous single-cell studies demonstrated that asynchronous, oscillatory RelA activities shape the late NF-κB response to TNF in WT cells (7, 12, 13). This oscillatory control was later implicated in NF-κB-driven gene expressions. Our bulk measurement based experimental analyses involving p100-deficient cells likely masked plausible cell-to-cell variations of the late-acting RelB:p50 response to TNFp. We argue that our study will further motivate single-cell analyses addressing the role of p100 in producing cellular heterogeneity at the level of NF-κB responses.

In sum, we show that brief TNF stimulation produces a long lasting RelB:p50 NF-κB activity in the absence of p100 that not only sustains the expression of a subset of RelA target, immune-response genes, but also activates genes with biological functions separable from immune responses. Stimulus-specific cellular responses are often achieved through distinct dynamical control of shared signaling kinases and transcription factors. For example, neuronal growth factor (NGF) induces the sustained activity of extracellular signal-regulated kinase (Erk) for promoting cell differentiation. In contrast, transient Erk activation by epidermal growth factor (EGF) causes cell proliferation (45, 46). Genome-wide knockdown studies indicated that a vast regulatory network, and not a handful of components belonging to specific pathways, controls the amplitude of the activity of these signaling molecules (47). The NF-κB system is comprised of interlinked canonical and non-canonical modules and controls the activity of multiple transcription factors, which have overlapping as well as distinct gene functions. Our study offered evidence that an interconnected NF-κB system, and not the individual NF-κB modules, directs dynamical activity of the specific NF-κB transcription factors in response to extracellular stimuli, and that the abundance of the immune-differentiation regulator p100 may inform cell-type specific biological responses to pro-inflammatory cytokines.

## Materials and Methods

### Mice, Cells, and Plasmids

WT and gene-deficient C57BL/6 mice were used in accordance with the guidelines of the Institutional Animal Ethics Committee of the National Institute of Immunology (approval no. #258/11). MEFs generated from E13.5 embryos were used subsequent to immortalization by the 3T3 protocol. Some key data have been also reproduced using primary MEFs. *Rela*^−/−^*Rel*^−/−^*Relb*^−/−^ 3T3 MEFs, which lacked the expression RelA, cRel, and RelB, were utilized as NF-κB-deficient cells. *Relb*^−/−^*Nfkb2*^−/−^ MEFs expressing transgenic RelB from retroviral constructs were reported earlier (23). Human-derived myeloma cell-lines OciMy5, KMS28PE, and JK6L used in this study were a kind gift from Dr. Michael Kuehl, NCI.

### Biochemical Analyses

In the TNFp regime, cells were treated briefly for 30 min with 1 ng/ml of TNF (Roche, Switzerland). Subsequently, TNF-supplemented media was substituted with TNF-free media, and cells were harvested at the indicated times after the commencement of the TNF treatment. In certain instances, cells were subjected to repeated pulses of TNF at the specified time intervals. Alternately, cells were treated chronically with 1 ng/ml of TNF (TNFc) or stimulated with 10 ng/ml IL-1β (Biosource, USA). As described (48), nuclear and whole cell extracts were analyzed by EMSA and Western blotting, respectively. The gel images were acquired using PhosphorImager (GE Amersham, UK) and quantified in ImageQuant 5.2.

### Gene Expression Analyses

Total RNA was isolated from cells, stimulated either briefly or chronically with 10 ng/ml of TNF, using RNeasy kit (Qiagen, Germany). qRT-PCR was performed as described earlier (23); see Table S1 for the description of primers. A detailed description of microarray mRNA analyses is available in the Supplementary Materials. The partition around medoid-based clustering analysis (49) was implemented in the Cluster package in R; the heatmap and violin plots were generated in MATLAB. For determining the significance of gene-expression differences between various genotypes within a given gene-group, we conducted multiple hypotheses testing and computed the effect sizes (Supplementary Materials). See Table S2 for a description of genes belonging to different gene-groups. The enrichment of the Gene Ontology terms was determined by Fisher's exact test using the “weight algorithm” available in topGO (50) and the entire Illumina MouseRef-8 v2.0 gene-array was used as the background. As described (51), ChIP experiments were performed using MEFs treated chronically with 10 ng/ml TNF (also see Supplementary Materials). Anti-RelA (sc-372) and anti-RelB antibodies (sc-226) were from Santa Cruz Biotechnology. Fold enrichment of RelA- or RelB-associated genes for a given gene group was computed against a list of randomly chosen 1,000 genes as control. We used Integrated Genome Viewer (IGV) to generate the browser tracks of individual genes.

### Computational Modeling

We utilized a previously published mass action kinetics-based NF-κB mathematical model (23) subsequent to necessary refinements (Supplementary Materials). These refinements improved the performance of the model with respect to the *Nfkbia*-deficient system, but preserved the model behavior observed earlier in WT and *Nfkb2*-deficient systems (23). The model was stimulated using Ode15s in MATLAB (2014b, Mathworks, USA). The abundances of various molecular species during early signaling was determined as the area under the respective timecourse curves between 0 and 2 h, and those during late signaling was estimated between 6 and 8 h. Variance-based, multiparametric sensitivity analysis has been described (29). Using iterative Monte Carlo sampling (1,000 simulations), we simultaneously explored a predetermined range (±10%) of parameter space around the initial values for the indicated parameter groups. The parameters belonging to a specific group were altered by the same factor for a given simulation.

See the Supplementary Materials and additional references (52–55) for the details of computational analyses on pathway modelling and microarray gene expression data.

### Statistical Analysis

Error bars were shown as S.E.M. of 3–6 experimental replicates. Quantified data are means ± SEM, and two-tailed Student's *t*-test was used for verifying statistical significance unless otherwise mentioned. Statistical tests associated with global gene-expression analyses have been detailed in the Supplementary Materials.

## Ethics Statement

WT and gene-deficient C57BL/6 mice were used in accordance with the animal usage guideline and recommendations of the Institutional Animal Ethics Committee of the National Institute of Immunology. The protocol was approved by the Institutional Animal Ethics Committee and the approval no is approval no. #258/11.

## Author Contributions

BC carried out *in silico* studies under the supervision of SB and JG. PR conducted cell-based analyses with the help from US, YR, and MC and the guidance from SB and RS. ChIP-seq experiments were performed by MZ and AS and analyzed by SD and BC with the guidance from RS and SB. BC and PR wrote the manuscript with SB.

### Conflict of Interest Statement

The authors declare that the research was conducted in the absence of any commercial or financial relationships that could be construed as a potential conflict of interest.

## References

[1] Kalliolias GD Ivashkiv LB. TNF biology, pathogenic mechanisms and emerging therapeutic strategies. Nat Rev Rheumatol. (2016) 12:49–62. 10.1038/nrrheum.2015.169 26656660 PMC4809675

[2] Mitchell S Vargas J Hoffmann A. Signaling via the NFkappaB system. Wiley Interdiscip Rev Syst Biol Med. (2016) 8:227–41. 10.1002/wsbm.1331 26990581 PMC8363188

[3] Beutler BA Milsark IW Cerami A. Cachectin/tumor necrosis factor: production, distribution, and metabolic fate *in vivo*. J Immunol. (1985) 135:3972–7. 2999236

[4] Hoffmann A Levchenko A Scott ML Baltimore D. The IkappaB-NF-kappaB signaling module: temporal control and selective gene activation. Science. (2002) 298:1241–5. 10.1126/science.1071914 12424381

[5] Cheong R Bergmann A Werner SL Regal J Hoffmann A Levchenko A. Transient IkappaB kinase activity mediates temporal NF-kappaB dynamics in response to a wide range of tumor necrosis factor-alpha doses. J Biol Chem. (2006) 281:2945–50. 10.1074/jbc.M510085200 16321974

[6] Werner SL Kearns JD Zadorozhnaya V Lynch C O'dea E Boldin MP. Encoding NF-kappaB temporal control in response to TNF: distinct roles for the negative regulators IkappaBalpha and A20. Genes Dev. (2008) 22:2093–101. 10.1101/gad.1680708 18676814 PMC2492747

[7] Nelson DE Ihekwaba AE Elliott M Johnson JR Gibney CA Foreman BE. Oscillations in NF-kappaB signaling control the dynamics of gene expression. Science. (2004) 306:704–8. 10.1126/science.1099962 15499023

[8] Tian B Nowak DE Brasier AR. A TNF-induced gene expression program under oscillatory NF-kappaB control. BMC Genomics. (2005) 6:137. 10.1186/1471-2164-6-137 16191192 PMC1262712

[9] Scott ML Fujita T Liou HC Nolan GP Baltimore D. The p65 subunit of NF-kappa B regulates I kappa B by two distinct mechanisms. Genes Dev. (1993) 7:1266–76. 10.1101/gad.7.7a.1266 8319912

[10] Lee EG Boone DL Chai S Libby SL Chien M Lodolce JP. Failure to regulate TNF-induced NF-kappaB and cell death responses in A20-deficient mice. Science. (2000) 289:2350–4. 10.1126/science.289.5488.2350 11009421 PMC3582399

[11] Kearns JD Basak S Werner SL Huang CS Hoffmann A. IkappaBepsilon provides negative feedback to control NF-kappaB oscillations, signaling dynamics, and inflammatory gene expression. J Cell Biol. (2006) 173:659–64. 10.1083/jcb.200510155 16735576 PMC2063883

[12] Ashall L Horton CA Nelson DE Paszek P Harper CV Sillitoe K. Pulsatile stimulation determines timing and specificity of NF-kappaB-dependent transcription. Science. (2009) 324:242–6. 10.1126/science.1164860 19359585 PMC2785900

[13] Paszek P Ryan S Ashall L Sillitoe K Harper CV Spiller DG. Population robustness arising from cellular heterogeneity. Proc Natl Acad Sci USA. (2010) 107:11644–9. 10.1073/pnas.0913798107 20534546 PMC2895068

[14] Hoffmann A. Immune response signaling: combinatorial and dynamic control. Trends Immunol. (2016) 37:570–2. 10.1016/j.it.2016.07.003 27461000 PMC5003693

[15] Sun SC. The noncanonical NF-kappaB pathway. Immunol Rev. (2012) 246:125–40. 10.1111/j.1600-065X.2011.01088.x 22435551 PMC3313452

[16] Derudder E Dejardin E Pritchard LL Green DR Korner M Baud V. RelB/p50 dimers are differentially regulated by tumor necrosis factor-alpha and lymphotoxin-beta receptor activation: critical roles for p100. J Biol Chem. (2003) 278:23278–84. 10.1074/jbc.M300106200 12709443

[17] Basak S Shih VF Hoffmann A. Generation and activation of multiple dimeric transcription factors within the NF-kappaB signaling system. Mol Cell Biol. (2008) 28:3139–50. 10.1128/MCB.01469-07 18299388 PMC2423155

[18] Lo JC Basak S James ES Quiambo RS Kinsella MC Alegre ML. Coordination between NF-kappaB family members p50 and p52 is essential for mediating LTbetaR signals in the development and organization of secondary lymphoid tissues. Blood. (2006) 107:1048–55. 10.1182/blood-2005-06-2452 16195333 PMC1895903

[19] Basak S Kim H Kearns JD Tergaonkar V O'dea E Werner SL. A fourth IkappaB protein within the NF-kappaB signaling module. Cell. (2007) 128:369–81. 10.1016/j.cell.2006.12.033 17254973 PMC1831796

[20] Shih VF Kearns JD Basak S Savinova OV Ghosh G Hoffmann A. Kinetic control of negative feedback regulators of NF-kappaB/RelA determines their pathogen- and cytokine-receptor signaling specificity. Proc Natl Acad Sci USA. (2009) 106:9619–24. 10.1073/pnas.0812367106 19487661 PMC2701028

[21] Tao Z Fusco A Huang DB Gupta K Young Kim D Ware CF. p100/IkappaBdelta sequesters and inhibits NF-kappaB through kappaBsome formation. Proc Natl Acad Sci USA. (2014) 111:15946–51. 10.1073/pnas.1408552111 25349408 PMC4234596

[22] Shih VF Davis-Turak J Macal M Huang JQ Ponomarenko J Kearns JD. Control of RelB during dendritic cell activation integrates canonical and noncanonical NF-kappaB pathways. Nat Immunol. (2012) 13:1162–70. 10.1038/ni.2446 23086447 PMC3634611

[23] Roy P Mukherjee T Chatterjee B Vijayaragavan B Banoth B Basak S. Non-canonical NFkappaB mutations reinforce pro-survival TNF response in multiple myeloma through an autoregulatory RelB:p50 NFkappaB pathway. Oncogene. (2017) 36:1417–29. 10.1038/onc.2016.309 27641334 PMC5346295

[24] Siggers T Chang AB Teixeira A Wong D Williams KJ Ahmed B. Principles of dimer-specific gene regulation revealed by a comprehensive characterization of NF-kappaB family DNA binding. Nat Immunol. (2011) 13:95–102. 10.1038/ni.2151 22101729 PMC3242931

[25] Zhao B Barrera LA Ersing I Willox B Schmidt SC Greenfeld H. The NF-kappaB genomic landscape in lymphoblastoid B cells. Cell Rep. (2014) 8:1595–606. 10.1016/j.celrep.2014.07.037 25159142 PMC4163118

[26] De Oliveira KA Kaergel E Heinig M Fontaine JF Patone G Muro EM. A roadmap of constitutive NF-kappaB activity in Hodgkin lymphoma: Dominant roles of p50 and p52 revealed by genome-wide analyses. Genome Med. (2016) 8:28. 10.1186/s13073-016-0280-5 26988706 PMC4794921

[27] Basak S Behar M Hoffmann A. Lessons from mathematically modeling the NF-kappaB pathway. Immunol Rev. (2012) 246:221–38. 10.1111/j.1600-065X.2011.01092.x 22435558 PMC3343698

[28] Le Novere N. Quantitative and logic modelling of molecular and gene networks. Nat Rev Genet. (2015) 16:146–58. 10.1038/nrg3885 25645874 PMC4604653

[29] Chatterjee B Banoth B Mukherjee T Taye N Vijayaragavan B Chattopadhyay S. Late-phase synthesis of IkappaBalpha insulates the TLR4-activated canonical NF-kappaB pathway from noncanonical NF-kappaB signaling in macrophages. Sci Signal. (2016) 9:ra120. 10.1126/scisignal.aaf1129 27923915 PMC5260935

[30] Li Q Brown JB Huang H Bickel PJ Measuring reproducibility of high-throughput experiments. Ann Appl Stat. (2011) 5:1752–79. 10.1214/11-AOAS466

[31] Covert MW Leung TH Gaston JE Baltimore D. Achieving stability of lipopolysaccharide-induced NF-kappaB activation. Science. (2005) 309:1854–7. 10.1126/science.1112304 16166516

[32] Werner SL Barken D Hoffmann A. Stimulus specificity of gene expression programs determined by temporal control of IKK activity. Science. (2005) 309:1857–61. 10.1126/science.1113319 16166517

[33] Adamson A Boddington C Downton P Rowe W Bagnall J Lam C. Signal transduction controls heterogeneous NF-kappaB dynamics and target gene expression through cytokine-specific refractory states. Nat Commun. (2016) 7:12057. 10.1038/ncomms12057 27381163 PMC4935804

[34] Roy P Sarkar UA Basak S. The NF-kappaB activating pathways in multiple myeloma. Biomedicines. (2018) 6:59. 10.3390/biomedicines6020059 29772694 PMC6027071

[35] Smale ST. Dimer-specific regulatory mechanisms within the NF-kappaB family of transcription factors. Immunol Rev. (2012) 246:193–204. 10.1111/j.1600-065X.2011.01091.x 22435556

[36] Zhao M Joy J Zhou W De S Wood WH III Becker KG. Transcriptional outcomes and kinetic patterning of gene expression in response to NF-kappaB activation. PLoS Biol. (2018) 16:e2006347. 10.1371/journal.pbio.2006347 30199532 PMC6147668

[37] Sedger LM Mcdermott MF. TNF and TNF-receptors: From mediators of cell death and inflammation to therapeutic giants - past, present and future. Cytokine Growth Factor Rev. (2014) 25:453–72. 10.1016/j.cytogfr.2014.07.016 25169849

[38] Vaira S Johnson T Hirbe AC Alhawagri M Anwisye I Sammut B. RelB is the NF-kappaB subunit downstream of NIK responsible for osteoclast differentiation. Proc Natl Acad Sci USA. (2008) 105:3897–902. 10.1073/pnas.0708576105 18322009 PMC2268780

[39] Tanaka S Nakano H. NF-kappaB2 (p100) limits TNF-alpha-induced osteoclastogenesis. J Clin Invest. (2009) 119:2879–81. 10.1172/JCI40629 19770519 PMC2752088

[40] Yao Z Xing L Boyce BF. NF-kappaB p100 limits TNF-induced bone resorption in mice by a TRAF3-dependent mechanism. J Clin Invest. (2009) 119:3024–34. 10.1172/JCI38716 19770515 PMC2752069

[41] Zhao Z Hou X Yin X Li Y Duan R Boyce BF. TNF Induction of NF-kappaB RelB enhances RANKL-induced osteoclastogenesis by promoting inflammatory macrophage differentiation but also limits it through suppression of NFATc1 expression. PLoS ONE. (2015) 10:e0135728. 10.1371/journal.pone.0135728 26287732 PMC4545392

[42] Courtois G Gilmore TD. Mutations in the NF-kappaB signaling pathway: implications for human disease. Oncogene. (2006) 25:6831–43. 10.1038/sj.onc.1209939 17072331

[43] Dang CV. Links between metabolism and cancer. Genes Dev. (2012) 26:877–90. 10.1101/gad.189365.112 22549953 PMC3347786

[44] Shaffer AL Emre NC Lamy L Ngo VN Wright G Xiao W. IRF4 addiction in multiple myeloma. Nature. (2008) 454:226–31. 10.1038/nature07064 18568025 PMC2542904

[45] Marshall CJ. Specificity of receptor tyrosine kinase signaling: transient versus sustained extracellular signal-regulated kinase activation. Cell. (1995) 80:179–85. 10.1016/0092-8674(95)90401-8 7834738

[46] Santos SD Verveer PJ Bastiaens PI. Growth factor-induced MAPK network topology shapes Erk response determining PC-12 cell fate. Nat Cell Biol. (2007) 9:324–30. 10.1038/ncb1543 17310240

[47] Friedman A Perrimon N. Genetic screening for signal transduction in the era of network biology. Cell. (2007) 128:225–31. 10.1016/j.cell.2007.01.007 17254958

[48] Banoth B Chatterjee B Vijayaragavan B Prasad MV Roy P Basak S. Stimulus-selective crosstalk via the NF-kappaB signaling system reinforces innate immune response to alleviate gut infection. Elife. (2015) 4:e05648. 10.7554/eLife.05648 25905673 PMC4432492

[49] Reynolds AP Richards G De La Iglesia B Rayward-Smith VJ Clustering rules: a comparison of partitioning and hierarchical clustering algorithms. J Math Model Alg. (2006) 5:475–504. 10.1007/s10852-005-9022-1

[50] Alexa A Rahnenfuhrer J Lengauer T. Improved scoring of functional groups from gene expression data by decorrelating GO graph structure. Bioinformatics. (2006) 22:1600–7. 10.1093/bioinformatics/btl140 16606683

[51] Heinz S Romanoski CE Benner C Allison KA Kaikkonen MU Orozco LD. Effect of natural genetic variation on enhancer selection and function. Nature. (2013) 503:487–92. 10.1038/nature12615 24121437 PMC3994126

[52] Authier H Billot K Derudder E Bordereaux D Riviere P Rodrigues-Ferreira S. IKK phosphorylates RelB to modulate its promoter specificity and promote fibroblast migration downstream of TNF receptors. Proc Natl Acad Sci USA. (2014) 111:14794–9. 10.1073/pnas.1410124111 25267645 PMC4205659

[53] Maitra R Melnykov V Assessing Significance in Finite Mixture Models. Technical Report, Department of Satistics, Iowa State University (2010).

[54] Mukherjee T Chatterjee B Dhar A Bais SS Chawla M Roy P. A TNF-p100 pathway subverts noncanonical NF-kappaB signaling in inflamed secondary lymphoid organs. EMBO J. (2017) 36:3501–16. 10.15252/embj.201796919 29061763 PMC5709727

[55] Sawilowsky SS New effect size rules of thumb. J Modern Appl Stat Methods. (2009) 8:597–99. 10.22237/jmasm/1257035100

