# Supplementary material for: Immune Differentiation Regulator p100 Tunes NF-κB Responses to TNF
Source: Front Immunol. 2019 May 7;10:997. doi: 10.3389/fimmu.2019.00997 (PMC6514058; doi:10.3389/fimmu.2019.00997)
Supplement: Supplementary file 1 [file Data_Sheet_1.docx]

***Supplementary Material***

**List of supplementary figures and tables**

Fig. S1. In silico studies of the NF-κB system Fig. S2. Analyzing TNF-induced NF-κB signaling in mutant cells Fig. S3. Investigating the mechanism underlying late-acting RelB:p50 response to TNFp in the absence of p100 Fig. S4. Investigating *Nfkb2*-deficient system in the repeated TNF pulse regime

Detailed description of global scale gene expression analyses Microarray mRNA analyses ChIP-Seq analyses

Fig. S5. Analyzing microarray data for determining the significance of gene-expression differences between various genotypes

Table S1. List of the primers used in our quantitative real-time PCR Table S2. A description of genes belonging to various gene-clusters and gene-groups presented in Figure 4.

Description of the mathematical model and related parameterization Fig. S6. Performance of the revised model *v2.0* in the TNFc regime Table S3. A list of biochemical constrains considered during model fitting. Table S4. A list of rate parameters subjected to modification. Fig. S7. Estimating the boundaries for the revised model Fig. S8. Performance of the Systems Model *v*2.1 in the TNFc regime Fig. S9. Comparing the performance of the NF-κB systems Model *v*2.1 with the previously published simulation data obtained using *v*2.0 Fig. S10. Examining the robustness of the conclusion with respect to the newly fitted parameter values Table S5. List of model parameters catalogued in 48 distinct groups


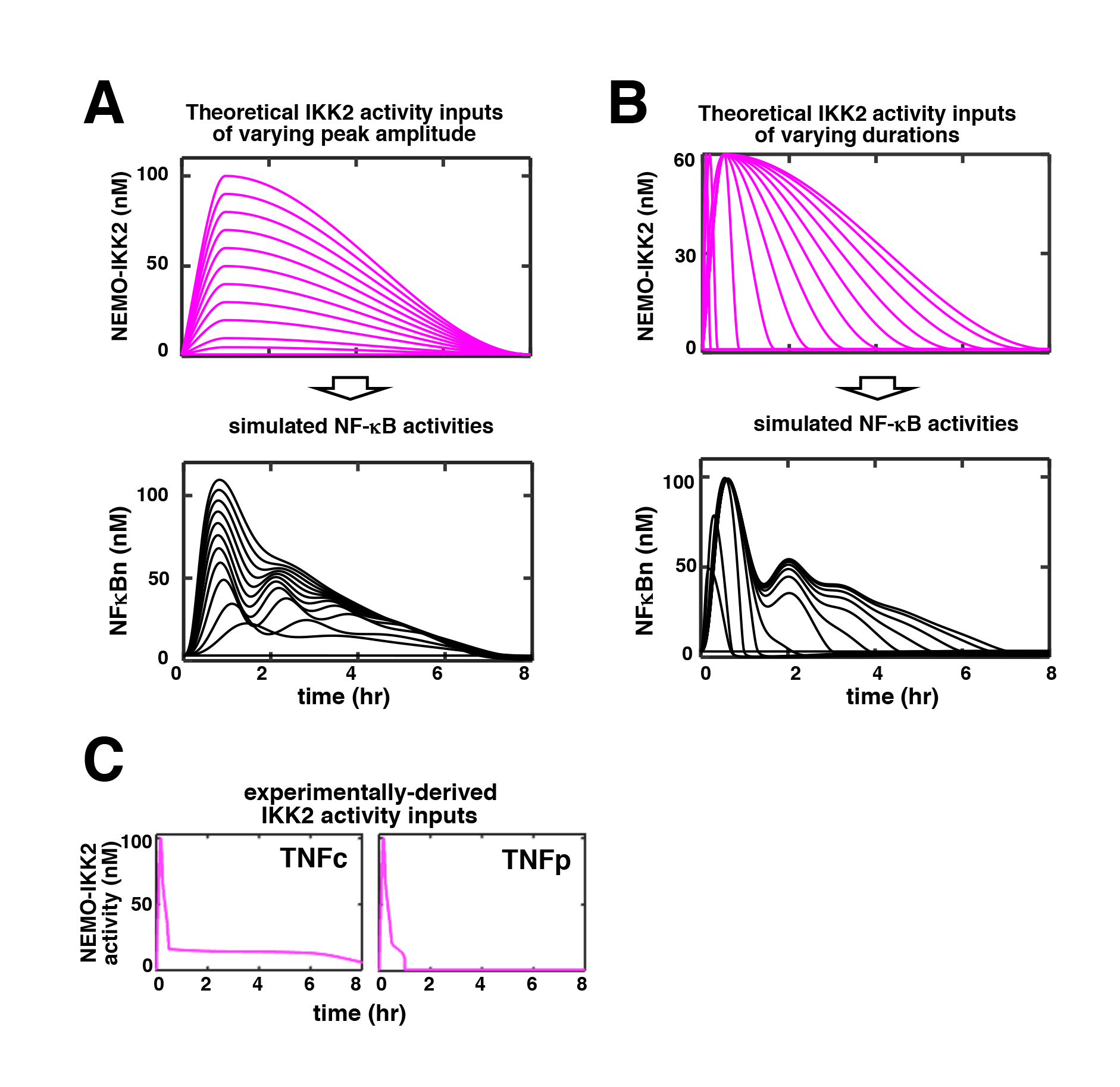


**Figure S1. In silico studies of the NF-κB system.** (**A**) A library of twelve theoretical IKK2 activity profiles with an invariant signal duration of 8 hr and peak amplitude uniformly varying from 10 nM to 100 nM was used as model inputs (top) for simulating NF-κBn responses in a time course (bottom). These IKK2 activity profiles had identical 1 hr onset time. (**B**) Thirteen IKK2 activity profiles with an identical 60 nM peak amplitude but total duration varying from 10 min to 480 min were similarly used in our simulation studies. We additionally used two IKK2 profiles with 60 nM peak amplitude, 5 min onset time and a total 10 min of duration or 60 nM peak amplitude, 10 min onset time and 20 min of duration as inputs. (**C**) Plots showing interpolated IKK2 activity curves generated using experimental data obtained from MEFs subjected to TNFc or TNFp stimulations (32).

**
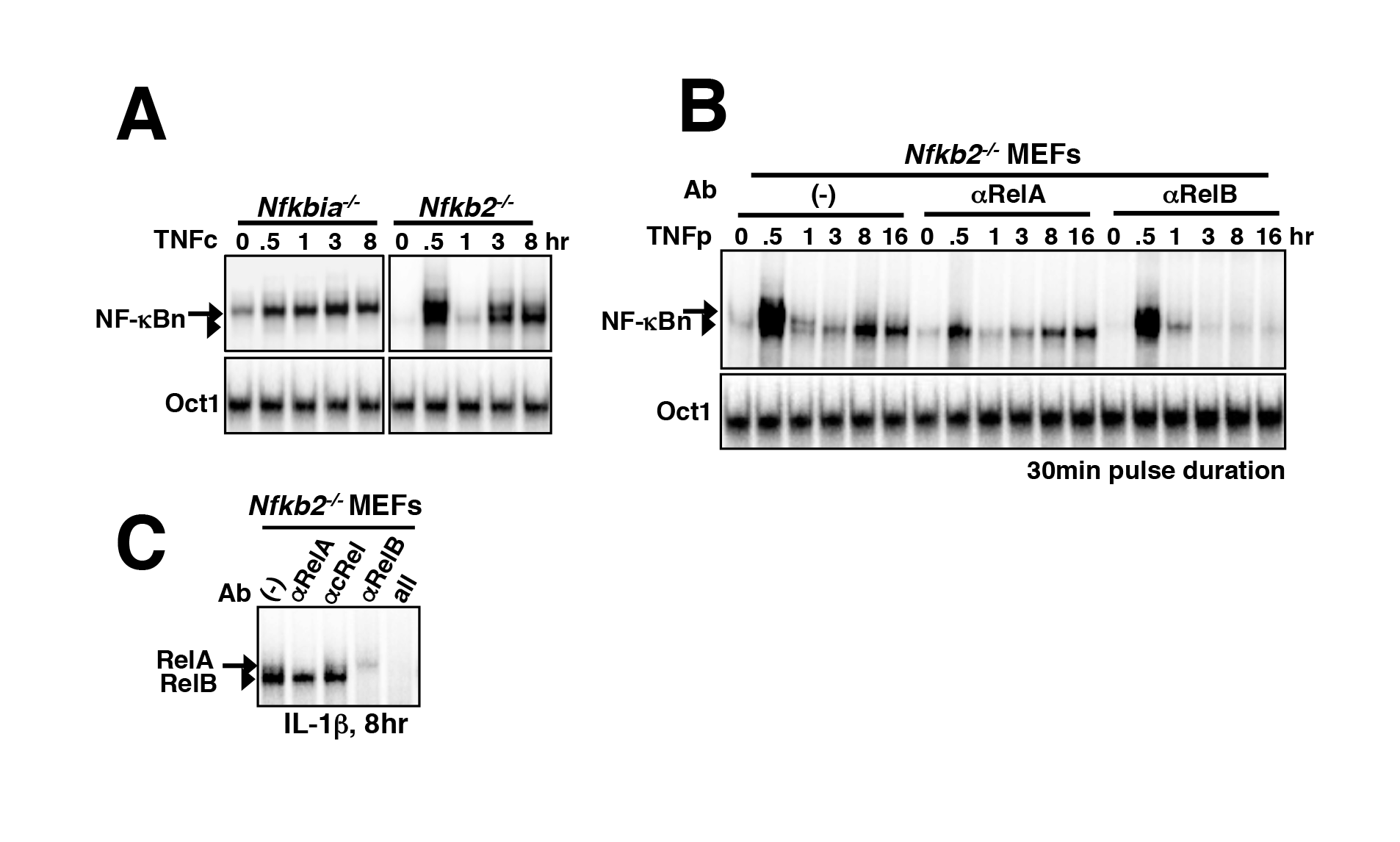
Figure S2. Analyzing TNF-induced NF-κB signaling in mutant cells. (A**) EMSA comparing NF-κBn induced in a time-course in *Nfkbia^-/-^* and *Nfkb2^-/-^* MEFs in response to TNFc. The data represents three independent experiments. (**B**) EMSA demonstrating total, RelB-containing and RelA-containing NF-κB activities induced in a time-course in response to TNFp. Dynamical RelB activity was unmasked in EMSA by ablating RelA DNA binding with an anti-RelA antibody. Similarly, RelA activity was unravelled by ablating RelB DNA binding. The data represents two biological replicates. (**C**) Composition of NF-κBn induced after 8 hr of IL-1β treatment in *Nfkb2^-/-^* MEFs was determined in the shift-ablation assay.

**
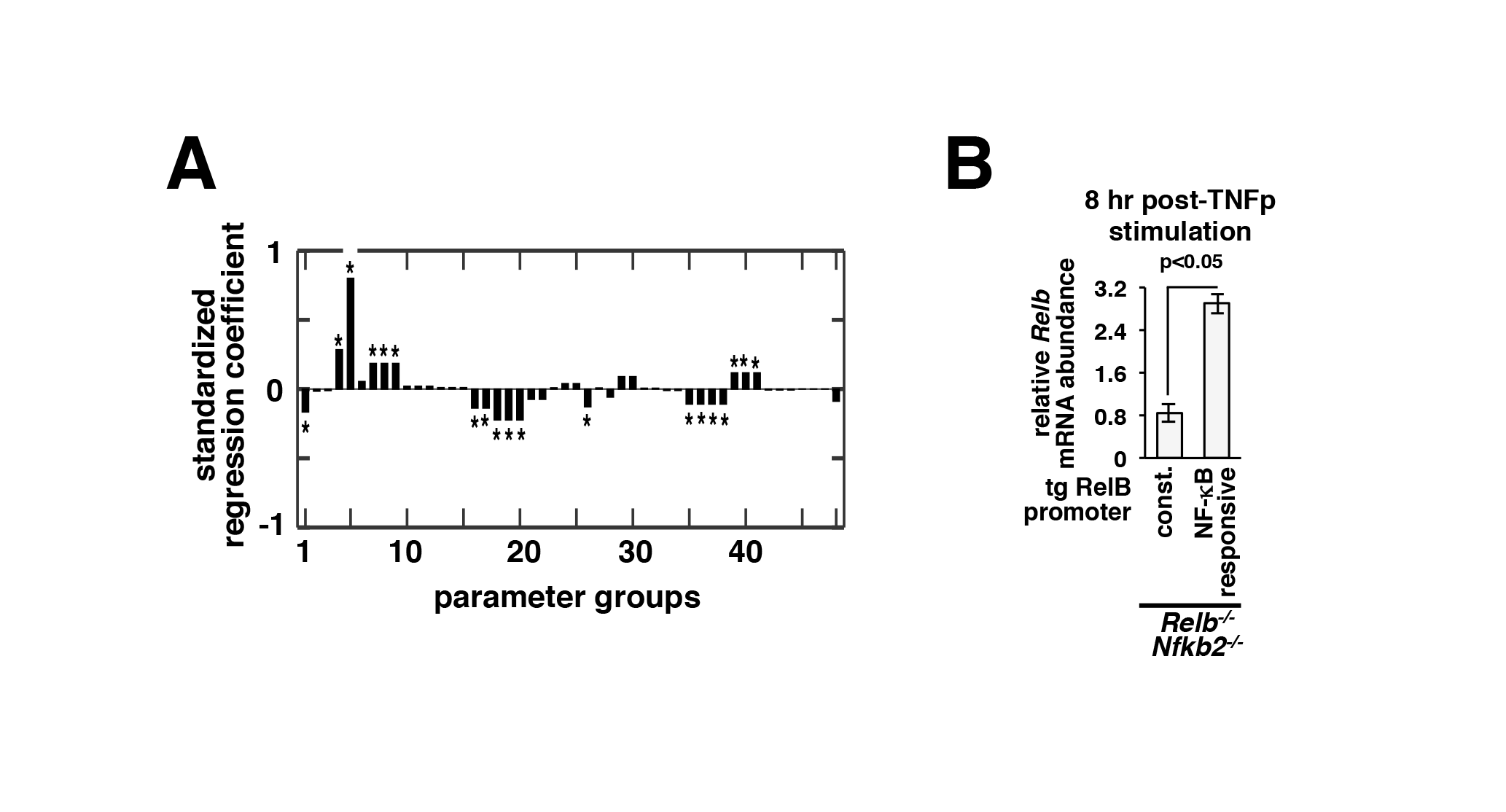
**

**Figure S3. Investigating the mechanism underlying late-acting RelB:p50 response to TNFp in the absence of p100. (A**) Graph plot describing the standardized regression coefficients of the parameter groups subjected to the Variance-based multiparametric sensitivity analysis. The asterisk indicates statistically significant deviation (*P* <0.001) from the null sensitivity for certain parameter groups, as determined by two-tailed Student’s t test. (**B**) qRT-PCR analysis revealing the expression of *Relb* mRNA after 8 hr of the commencement of TNFp treatment in *Relb^-/-^Nfkb2^-/-^* MEFs stably expressing RelB from a transgene (tg) either constitutively (const.) or from an NF-κB responsive promoter (top panel). Data are means ± SEM of four biological replicates.

**
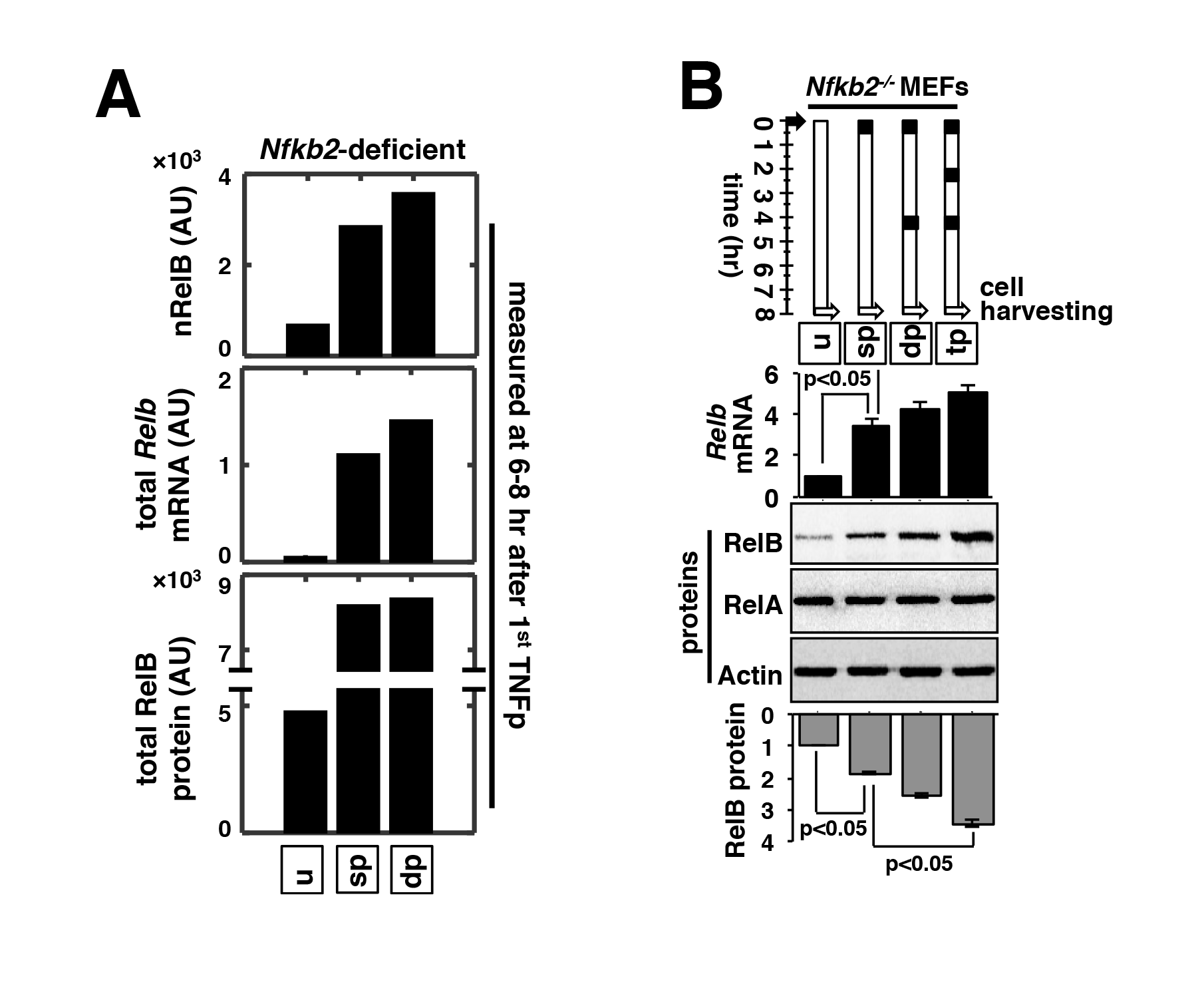
**

**Figure S4. Investigating *Nfkb2*-deficient system in the repeated TNF pulse regime.** (**A**) Computational simulation studies predicting nRelB activity (top panel), abundances of *Relb* mRNA as well as RelB protein in *Nfkb2*-deficient system subjected to a single TNFp (sp) or successive two TNFp separated by 4 hr (dp). The activities and abundances were estimated at 6-8 hr after the commencement of the first pulse. u denote untreated system. (**B**) *Nfkb2^-/-^* MEFs were treated with either a single TNFp (single pulse, sp) or two successive TNFp separated by 4 hr (double pulse, dp) or three successive TNFp where these pulses were separated by 2 hr (tp). Cells were harvested 8 hr after the commencement of the first pulse and subjected to either qRT-PCR analyses of *Relb* mRNA abundances (top bargraph) or Western Blotting analysis with antibodies against the indicated proteins (bottom panels). Densitometric analysis of the relative abundances of RelB protein has been also presented in a bargraph (bottom). mRNA data are means ± SEM of four biological replicates, protein data are means ± SEM of three experimental repeats.

**Detailed description of global scale gene expression analyses**

**Microarray mRNA analyses**

For microarray mRNA analyses, labelling, hybridization to the Illumina MouseRef-8 v2.0 Expression BeadChip, data processing, and quantile normalization were performed by Sandor Pvt Ltd (Hyderabad, India). We used a rank-based method for selecting genes with essentially zero detection p-value (30). Among these reproducibly expressed genes, we further considered genes whose expressions were induced at least 1.3 fold at upon 6 hr of TNFc treatment in *Nfkb2^-/-^* MEFs, but not in NF-κB-deficient cells. Our analyses led us to a list of 304 NF-κB-dependent genes. Subsequently, we utilized the partition around medoid-based algorithm, which allows for robust clustering of datasets with significant outliers (Reynolds et al., 2006). In the partition around medoid-based clustering analysis, typically the Manhattan distances, and not the Euclidean distances, between the data points are calculated. Nevertheless, we utilized the Cluster package present in R for clustering analysis and the heatmap as well as violin plots were generated in MATLAB. As indicated in the main text, we catalogued 304 NF-κB-dependent genes into six distinct clusters, which were arranged further into four gene-groups. The significance of gene-expression differences between various genotypes within a given gene-group was determined by combining the multiple hypotheses testing method with a stringent false discovery correction strategy (53). We implemented individually right and left tailed t-tests for false discovery rate < 0.001, and examined if the mean of a distribution was significantly different than the mean of another distribution. The numerical significance of the difference in the mean of two distributions was captured from the effect size (Cohen’s d). The data was visualized using the q-value map (Fig. S5), as described earlier (55).

**ChIP-seq analyses**

ChIP experiments were performed as described (51). Briefly, cells were washed twice with cold PBS and then were fixed at room temperature with 1.5mM EGS (Pierce Cat # 21565) in PBS for 30 min followed by 1% formaldehyde (Sigma-Aldrich) for 15 min. Crosslinking reactions were quenched using 0.125M glycine, and cells were washed twice. Subsequently, nuclei were isolated and lysed in a buffer containing 50mM Hepes-KOH, pH 7.5; 150mM NaCl; 1mM EDTA; 1% Triton X-100; 0.1% sodium deoxycholate; 0.1% SDS and protease inhibitors. The crosslinked chromatin was subjected to fragmentation by sonication (Branson Sonicator). Chromatin immunoprecipitation (ChIP) was performed using anti-RelA antibody (Santa Cruz, sc-372) or anti-RelB antibody (sc-226) bound to 50μl Protein A Dynabeads (Invitrogen). Beads were collected by centrifugation, washed, and incubated at 65°C for 4h in the elution buffer (50mM Tris-HCl, pH 7.5; 10mM EDTA; 1% SDS) for reverse crosslinking. ChIP DNA was purified by phenol-chloroform extraction followed by ethanol precipitation.

ChIP seq library was prepared using Nugen Ovation Ultra-low library preparation kit *v.2* (Nugen, San Carlo, CA). The libraries were sequenced on Illumina NextSeq 500 for single end reads. Single-end reads were demultiplexed, adapters were removed by cutadapt and bad quality bases (Q<20) were removed. The cleaned FASTQ files were aligned against mm9 genome using Bowtie with "--best --strata" options and unique alignments were reported. Peaks were called using cisGenome (v2.0) using a window size of 200 bases and a cutoff of 4 reads in total (2 reads from each strand), FDR<0.1 and Fold change >2. Inputs from WT or *Nfkb2^-/-^* MEFs were used for analyzing corresponding ChIP-seq samples. The data was annotated using Ensembl mm9 v67 genome annotation files. For assigning peaks to genes, we considered NF-κB binding to chromatin locations <50kb away from the transcription start site of a given gene. The Chip-seq data set is available on NCBI-GEO (accession no. GSE119961).

**Fig S5. Analyzing microarray data for determining the significance of gene-expression differences between various genotypes.** We subjected our data set to right and left tailed t-tests, considering false discovery rate < 0.001, and examined if the mean of a pair of distributions was significantly different. We also determined the effect size as a measure of the numerical significance of these differences. The data has been presented using q-value maps. Absence of a block in a q-value map indicates insignificant difference between a pair of distributions. In other words, it signifies that the expression of genes belonging to a given groups is not significantly different between the indicated pair of genotypes. The color bar captures effect sizes for statistically significant pairs.

**Table S1. List of the primers used in our quantitative real-time PCR.**

**Table S2. A description of genes belonging to various gene-clusters and gene-groups presented in Figure 4.**

**Description of the mathematical model and related parameterization**

In our previously published mathematical model *v2.0* (23), we depicted signal-responsive activation of four NF-κB heterodimers, namely RelA:p50, RelA:p52, RelB:p50 and RelB:p52. Combinatorial association of RelA, RelB, p50, and p52 produced these heterodimers. Except for RelB:p52, these heterodimers were sequestered in the unstimulated system by IκBα, IκBβ, IκBε or IκBδ/(p100)_2_. IκBβ did not interact with RelB:p50. Experimentally measured or theoretical NEMO-IKK2 and NIK-IKK1 activity profiles were used as model inputs. Signal-induced degradation of inhibitory proteins led to nuclear translocation of the bound NF-κB heterodimers. On the other hand, RelB:p52 was produced upon preferential binding of RelB to p52, which was generated from p100 in response to noncanoncial signals. Once generated, RelB:p52 translocated into the nucleus. RelB also bound to p100, but the resultant RelB:p100 complex was unresponsive to canonical or noncanonical signals. As such, RelA, p50 and IκBβ were produced involving constitutive transcription reaction. Synthesis of RelB, p100/*Nfkb2*, IκBα and IκBε involved both NF-κB-independent (constitutive) as well as dependent transcriptions. Because of a lack of experimental evidence, we restricted RelB:p52 from mediating the expression of genes encoding NF-κB/IκB proteins in this model.

We recently observed that IκBδ/(p100)_2_ also sequesters RelB:p52 (54). Accordingly, we revised the model *v2.0* to include the description of IκBδ/(p100)_2_-mediated inhibition of RelB:p52. The corresponding rate parameters were considered to be identical to those associated with RelB:p50 and IκBδ/(p100)_2_ interaction.

Next, it was shown that IKK2 phosphorylates RelB during TNF signaling and that IKK2-mediated phosphorylation prevents RelB:p50 binding to IκBα (52). In the model *v2.0*, we assumed that TNF converts pre-existing RelB or RelB:p50 into respective RelB* or RelB*p50. As such RelB and RelB* possessed identical properties except that RelB*p50 bound to IκBα and IκBε with ~100-fold lower affinities. The conversion of RelB into RelB* occurred throughout the 8 hr of the TNFc regime. Here, we further revised the model and elaborately described RelB* generation that allowed us to simulate both chronic and brief TNF treatment regimes. We first included the description of RelB*:p52, whose properties were similar to that of RelB:p52. We permitted the conversion of RelB and RelB heterodimers (RelB:p50 and RelB:p52) into corresponding RelB* and RelB* heterodimers for 8h for the TNFc regime or for stimulation regimes with the duration of the IKK2 activity ≥ 4h. For brief TNF regimes with the duration of the IKK2 activity <4h, conversion started along with the onset of TNF signaling but continued for a time period that was double the duration of the TNF-induced transient IKK2 activity. We computed the duration of the input as the time elapsed above an arbitrarily defined threshold concentration of 15nM in the corresponding kinase activity curve.

Furthermore, we assumed that only RelB*:p50, and not RelB:p50, participated in the autoregulatory RelB synthesis. This assumption was consistent with the notion that TNF modification enhanced the transcriptional activity of RelB. Finally, we terminated RelA-mediated transcription of RelB at 2 h post-TNF stimulation; this was terminated at 1.5h post-TNF treatment in the original *v2.0*.

Using this revised model, we attempted to recapitulate *in silico* experimental TNF signaling, whose dynamical control is thought to be determined by IκBα. Our simulation studies revealed elevated RelA as well as RelB activities in the IκBα-deficient system (Fig. S6). Of note, experimental analyses indicated a substantially heightened nuclear RelA, but not RelB, activity in IκBα-null cells (Roy et al., 2017). To resolve this discrepancy, we further manually refined the parameter ensemble. We considered eighteen, experimentally-derived, quantifiable constrains for the parameterization exercise (Table S3). Together, we altered sixteen rate parameters to arrive onto the model *v2.1* (Table S4)*,* which satisfied all the pre-determined constraints. Actual changes in the parameter values in *v2.1* were subtle and mostly within the range of ~ 2-5 fold.

**Fig. S6. Performance of the revised model *v2.0* in the TNFc regime:** Simulation of the previously published *(21)* mathematical model revealing TNFc-induced NF-κB activity in WT and *Nfkbia*-deficient systems. Activities of RelA and RelB heterodimers have been indicated.

Importantly, the model *v2.1* satisfied the biochemical constraints even upon a ~ 1.5-10 fold increase or decrease in the newly-fitted parameter values (Fig. S7). These analyses suggested that the model *v2.1* possessed a modestly broad boundary.

**Table S3. A list of biochemical constrains considered during model fitting.**

**Table S4. A list of rate parameters subjected to modification.**

**Fig. S7. Estimating the boundaries for the revised model.** A set of sixteen rate parameters were subjected to revisions to arrive onto the NF-κB system model *v*2.1. We then simulated the model *v*2.1 iteratively using various multipliers (0.1, 0.2, 0.5, 1, 2, 5, and 10) for these parameters. Following each simulation, the model was compared against the list of eighteen established constraints presented in table S3. The bars show the maximum and minimum multipliers for each of the rate constant groups that satisfy all the constraints.

When we simulated TNF signaling in the IκBα–deficient system using *v2.1*, we indeed noticed an improvement in the model performance. Consistent with experimental results, the model *v2.1* revealed a heightened nuclear RelA activity and a low-level of nuclear RelB activity in the absence of IκBα (Fig. S8). Prior simulation studies involving the *v2.0* led to the discovery of an autoregulatory RelB pathway (23). The model *v2.1* aptly reproduced key data published earlier by Roy et al., using *v2.0* (Fig. S9). Our current study indicated that RelB synthesis acted as a key determinant of the late RelB activity induced upon brief TNF stimulation of p100-deficient cells. We tested if the newly-fitted parameters influenced our overall conclusion. To this end, we incrementally increased these rate parameters up to 4 fold or similarly decreased them. Subsequently, we scored the effect of parameter perturbations on the late RelB activity induced in the p100-deficient system. Our analyses assured that with the expected exception of the RelB synthesis related rate parameters (#3, #11 and #15), the majority of the newly-fitted parameters had only subtle impact on the late nuclear RelB activity (Fig. S10). Nevertheless, the final model *v2.1* consisted of 77 species, 296 reactions and 214 parameters. We used MATLAB 2014b for designing, simulating the model and for the generation of the figures.

**Fig. S8. Performance of the Systems Model *v*2.1 in the TNFc regime.** Simulation of the revised NF-κB System model *v*2.1 revealing TNFc-induced NF-κB activity in WT and IκBα-deficient systems. Activities of RelA and RelB heterodimers have been indicated.

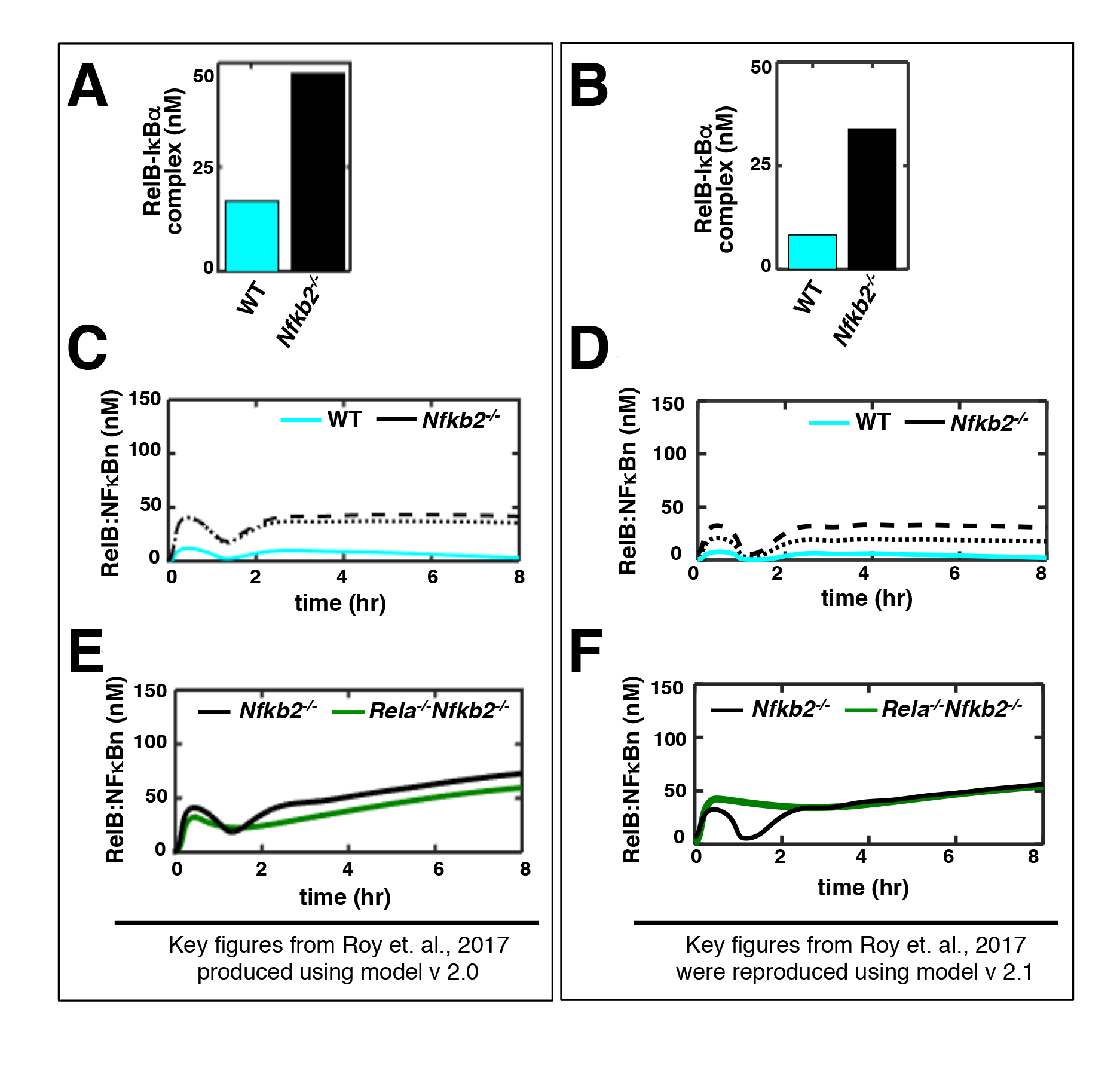


**Fig S9. Comparing the performance of the NF-κB systems Model *v*2.1 with the previously published simulation data obtained using *v*2.0.** Computational simulations using the previously published NF-κB systems Model *v*2.0 (**A**, **C** and **E**) and the model *v*2.1(**B**, **D** and **F**). We compared RelB binding to IκBα in WT and *Nfkb2*-deficient system (**A** and **B**), and dynamical RelB activation in various knockout systems (**C**-**F**) in these two model versions.

**Fig S10. Examining the robustness of the conclusion with respect to the newly fitted parameter values.** The values of each of the sixteen newly fitted rate parameters in the model *v2.1* were increased incrementally up to 4 fold (blue circles) or decreased similarly up to 4 fold (red circles). The resulting parameter ensembles were used for simulating the TNFp regime in the *Nfkb2*-deficient system, and the late nRelB activities were estimated. The horizontal line parallel to the X-axis represents the late nRelB activity induced by TNFp in the *Nfkb2*-defiicent system using the unaltered parameter values.

**Table S5. List of model parameters catalogued into 48 distinct groups.**

Below we provide the complete list of model parameters catalogued into 48 groups, as described in Figure 3a.

| **Param No.** | **Reaction** | **Parameter Value** | **Category** | **Unit** | **Location** | **Group No.** |
| --- | --- | --- | --- | --- | --- | --- |
| 001 | 🡺 tIkBa | 3.0e-3 | RNA synthesis | nM.min^-1^ | --- | **Group I** |
|  |  |  | constitutive transcription rate |  |  |  |
| 002 |  | 25 | Maximum expression fold change induced by RelA:p50 dimers (k_d_ = 45 nM) | - |  |  |
| 024 | 🡺 IkBa | 12 | Protein synthesis | min^-1^ | cytoplasm |  |
| 004 | 🡺 tIkBb | 6.6e-4 | RNA Synthesis | nM.min^-1^ | --- | **Group II** |
|  |  |  | constitutive transcription rate |  |  |  |
| 025 | 🡺 IkBb | 12 | Protein synthesis | min^-1^ | cytoplasm |  |
| 005 | 🡺 tIkBe | 6.0e-5 | RNA Synthesis | nM.min^-1^ | --- | **Group III** |
|  |  |  | constitutive transcription rate |  |  |  |
| 006 |  | 20 | Maximum expression fold change induced by RelA:p50 dimers (k_d_ = 50 nM) | - |  |  |
| 026 | 🡺 IkBe | 12 | Protein synthesis | min^-1^ | cytoplasm |  |
| 008 | 🡺 tp100 | 4.7e-5 | RNA Synthesis | nM.min^-1^ | --- | *****  Excluded from the analysis, since *Nfkb2* null system was investigated. |
|  |  |  | constitutive transcription rate |  |  |  |
| 009 |  | 33 | Maximum expression fold change induced by RelA:p50 dimers (k_d_ = 45 nM) | - |  |  |
| 027 | 🡺 p100 | 12 | Protein synthesis | min^-1^ | cytoplasm |  |
| 011 | 🡺 tRelA | 1.2e-5 | RNA Synthesis  (constitutive) | nM.min^-1^ | --- | **Group IV** |
| 028 | 🡺 RelA | 12 | Protein synthesis | min^-1^ | cytoplasm |  |
| 012 | 🡺 tRelB | 1.125e-5 | RNA Synthesis | nM.min^-1^ | --- | **Group V** |
|  |  |  | constitutive transcription rate |  |  |  |
| 013 |  | 11 | Maximum expression fold change induced by RelA:p50 and RelB*:p50 dimers (k_d_ = 10 nM) | - |  |  |
| 029 | 🡺 RelB | 12 | Protein synthesis | min^-1^ |  |  |
| 016 | 🡺 tp50 | 1.0e-4 | RNA Synthesis  (constitutive) | nM.min^-1^ | --- | **Group VI** |
| 030 | 🡺 p50 | 12 | Protein synthesis | min^-1^ | cytoplasm |  |
| 017 | tIkBa 🡺 | 3.2e-2 | RNA degradation | min^-1^ | --- | **Group VII** |
| 031 | IkBa 🡺 | .06 | Protein degradation | min^-1^ | cytoplasm& nucleus |  |
| 041 | IkBa + NEMO-IKK 🡺 | 1.4e-3 | NEMO mediated free IκB degradation | nM^-1^.min^-1^ | cytoplasm |  |
| 018 | tIkBb 🡺 | 6.9e-3 | RNA degradation | min^-1^ | --- | **Group VIII** |
| 032 | IkBb 🡺 | .18 | Protein degradation | min^-1^ | cytoplasm& nucleus |  |
| 042 | IkBb + NEMO-IKK 🡺 | 4.5e-4 | NEMO mediated free IκB degradation | nM^-1^.min^-1^ | cytoplasm |  |
| 019 | tIkBe 🡺 | 3.8e-3 | RNA degradation | min^-1^ | --- | **Group IX** |
| 033 | IkBe 🡺 | .18 | Protein degradation | min^-1^ | cytoplasm& nucleus |  |
| 043 | IkBe + NEMO-IKK 🡺 | 9e-4 | NEMO mediated free IκB degradation | nM^-1^.min^-1^ | cytoplasm |  |
| 020 | tp100 🡺 | 1.9e-3 | RNA degradation | min^-1^ | --- | ***** |
| 035 | P100 🡺 | 5.7e-3 | Protein degradation | min^-1^ | cytoplasm& nucleus |  |
| 034 | IkBd 🡺 | 1.1e-2 | Protein degradation | min^-1^ | cytoplasm& nucleus |  |
| 044 | IkBd + NIK-IKK1 🡺 | 1.0e-3 | NIK mediated free IκBδ degradation | nM^-1^.min^-1^ | cytoplasm |  |
| 021 | tRelA 🡺 | 2.9e-3 | RNA degradation | min^-1^ | --- | **Group X** |
| 036 | RelA 🡺 | 5.7e-3 | Protein degradation | min^-1^ | cytoplasm& nucleus |  |
| 022 | tRelB 🡺 | 9.2e-3 | RNA degradation | min^-1^ | --- | **Group XI** |
| 037 040 | RelB 🡺 RelB* 🡺 | 5.7e-3 | Protein degradation | min^-1^ | cytoplasm& nucleus |  |
| 023 | tp50 🡺 | 2.9e-3 | RNA degradation | min^-1^ | --- | **Group XII** |
| 038 | p50 🡺 | 3.7e-3 | Protein degradation | min^-1^ | cytoplasm& nucleus |  |
| 053 | RelA + p50 🡺 RelA:p50 | 9.6e-4 | monomer association | nM^-1^.min^-1^ | cytoplasm& nucleus | **Group XIII** |
| 055 | RelB + p50 🡺 RelB:p50 | 9.6e-4 | monomer association | nM^-1^.min^-1^ | cytoplasm& nucleus | **Group XIV** |
| 058 | RelB* + p50 🡺RelB*:p50 | 9.6e-4 | monomer association | nM^-1^.min^-1^ | cytoplasm& nucleus |  |
| 093 | RelA:p50 + IkBa 🡺 RelA:p50:IkBa | 3.6e-1 | association | nM^-1^.min^-1^ | cytoplasm& nucleus | **Group XV** |
| 094 | RelA:p50 + IkBb 🡺 RelA:p50:IkBb | 2.0e-1 | association | nM^-1^  min^-1^ | cytoplasm& nucleus | **Group XVI** |
| 095 | RelA:p50 + IkBe 🡺 RelA:p50:IkBe | 2.0e-1 | association | nM^-1^.min^-1^ | cytoplasm& nucleus | **Group XVII** |
| 054 | RelA + p52 🡺 RelA:p52 | 9.6e-4 | monomer association | nM^-1^.min^-1^ | cytoplasm & nucleus | ***** |
| 096 | RelA:p50 + IkBd 🡺 RelA:p50:IkBd | 8.0e-2 | association | nM^-1^.min^-1^ | cytoplasm& nucleus |  |
| 097 | RelA:p52 + IkBa 🡺 RelA:p52:IkBa | 2.0e-1 | association | nM^-1^.min^-1^ | cytoplasm& nucleus |  |
| 098 | RelA:p52 + IkBb 🡺 RelA:p52:IkBb | 1.0e-1 | association | nM^-1^.min^-1^ | cytoplasm& nucleus |  |
| 099 | RelA:p52 + IkBe 🡺 RelA:p52:IkBe | 1.0e-1 | association | nM^-1^.min^-1^ | cytoplasm& nucleus |  |
| 100 | RelA:p52 + IkBd 🡺 RelA:p52:IkBd | 8.0e-2 | association | nM^-1^.min^-1^ | cytoplasm& nucleus |  |
| 101 | RelB:p50 + IkBa 🡺 RelB:p50:IkBa | 9.0e-2 | association | nM^-1^.min^-1^ | cytoplasm& nucleus | **Group XVIII** |
| 104 | RelB*:p50 + IkBa 🡺 RelB*:p50:IkBa | 9.0e-3 | association | nM^-1^.min^-1^ | cytoplasm& nucleus |  |
| 102 | RelB:p50 + IkBe 🡺 RelB:p50:IkBe | 4.0e-2 | association | nM^-1^.min^-1^ | cytoplasm& nucleus | **Group XIX** |
| 105 | RelB*:p50 + IkBe 🡺 RelB*:p50:IkBe | 4.0e-3 | association | nM^-1^.min^-1^ | cytoplasm& nucleus |  |
| 056 | RelB + p52 🡺 RelB:p52 | 9.6e-4 | monomer association | nM^-1^.min^-1^ | cytoplasm& nucleus | ***** |
| 060 | RelB* + p52 🡺 RelB*:p52 | 9.6e-4 | monomer association | nM^-1^.min^-1^ | cytoplasm& nucleus |  |
| 057 | RelB + p100 🡺RelB:p100 | 5.7e-2 | monomer association | nM^-1^  min^-1^ | cytoplasm& nucleus |  |
| 059 | RelB* + p100 🡺 RelB*p100 | 5.7e-2 | monomer association | nM^-1^.min^-1^ | cytoplasm& nucleus |  |
| 103 | RelB:p50 + IkBd 🡺 RelB:p50:IkBd | 1.2e-1 | association | nM^-1^.min^-1^ | cytoplasm& nucleus |  |
| 106 | RelB*:p50 + IkBd 🡺 RelB*:p50:IkBd | 1.2e-1 | association | nM^-1^.min^-1^ | cytoplasm& nucleus |  |
|  |  |  |  |  |  |  |
| 107 | RelB:p52 + IkBd 🡺 RelB:p52:IkBd | 1.2e-1 | association | nM^-1^.min^-1^ | cytoplasm& nucleus |  |
| 108 | RelB*:p52 + IkBd 🡺 RelB*:p52:IkBd | 1.2e-1 | association | nM^-1^.min^-1^ | cytoplasm& nucleus |  |
| 061 | RelA:p50 🡺 RelA + p50 | 1.4e-3 | dimer dissociation | min^-1^ | cytoplasm& nucleus | **Group XX** |
| 085 | RelA:p50 🡺 | 2.4e-4 | protein degradation | min^-1^ | cytoplasm& nucleus |  |
| 063 | RelB:p50 🡺 RelB + p50 | 1.4e-3 | dimer dissociation | min^-1^ | cytoplasm& nucleus | **Group XXI** |
| 066 | RelB*:p50 🡺 RelB* + p50 | 1.4e-3 | dimer dissociation | min^-1^ | cytoplasm& nucleus |  |
| 087 | RelB:p50 🡺 | 2.4e-4 | protein degradation | min^-1^ | cytoplasm& nucleus |  |
| 090 | RelB*:p50 🡺 | 2.4e-4 | protein degradation | min^-1^ | cytoplasm& nucleus |  |
| 062 | RelA:p52 🡺 RelA + p52 | 1.4e-3 | dimer dissociation | min^-1^ | cytoplasm& nucleus | ***** |
| 086 | RelA:p52 🡺 | 2.4e-4 | protein degradation | min^-1^ | -do- |  |
| 064 | RelB:p52 🡺 RelB + p52 | 1.4e-3 | dimer dissociation | min^-1^ | -do- | ***** |
| 065 | RelB:p100 🡺 RelB + p100 | 1.4e-3 | dimer dissociation | min^-1^ | -do- |  |
| 067 | RelB*:p100🡺 RelB* + p100 | 1.4e-3 | dimer dissociation | min^-1^ | -do- |  |
| 088 | RelB:p52 🡺 | 2.4e-4 | protein degradation | min^-1^ | -do- |  |
| 092 | RelB*:p52n 🡺 | 2.4e-4 | protein degradation | min^-1^ | -do- |  |
| 089 | RelB:p100 🡺 | 2.5e-3 | protein degradation | min^-1^ | -do- |  |
| 091z | RelB*:p100🡺 | 2.5e-3 | protein degradation | min^-1^ | cytoplasm& nucleus |  |
| 109 | RelA:p50:IkBa 🡺 RelA:p50 + IkBa | 8.4e-3 | dissociation | min^-1^ | cytoplasm& nucleus | **Group XXII** |
| 125 | RelA:p50:IkBa 🡺 RelA:p50 | 2e-4 | constitutive protein degradation | min^-1^ | cytoplasm& nucleus |  |
| 157 | RelA:p50:IkBa + NEMO 🡺RelA:p50 | 1.4e-3 | NEMO mediated protein degradation | nM^-1^.min^-1^ | cytoplasm |  |
| 110 | RelA:p50:IkBb🡺  RelA:p50 + IkBb | 3.4e-2 | dissociation | min^-1^ | cytoplasm& nucleus | **Group XXIII** |
| 126 | RelA:p50:IkBb 🡺 RelA:p50 | 2e-4 | constitutive protein degradation | min^-1^ | cytoplasm& nucleus |  |
| 158 | RelA:p50:IkBb + NEMO 🡺RelA:p50 | 4.5e-4 | NEMO mediated protein degradation | nM^-1^.min^-1^ | cytoplasm |  |
| 111 | RelA:p50:IkBe 🡺 RelA:p50 + IkBe | 8.4e-3 | dissociation | min^-1^ | cytoplasm& nucleus | **Group XXIV** |
| 127 | RelA:p50:IkBe 🡺 RelA:p50 | 2e-4 | constitutive protein degradation | min^-1^ | cytoplasm& nucleus |  |
| 159 | RelA:p50:IkBe + NEMO 🡺RelA:p50 | 9.0e-4 | NEMO mediated protein degradation | nM^-1^.min^-1^ | cytoplasm |  |
| 112 | RelA:p50:IkBd 🡺 RelA:p50 + IkBd | 1.7e-2 | dissociation | min^-1^ | cytoplasm& nucleus | ***** |
| 128 | RelA:p50:IkBd 🡺 RelA:p50 | 2e-4 | constitutive protein degradation | min^-1^ | cytoplasm& nucleus |  |
| 167 | RelA:p50:IkBd + NIK-IKK1 🡺RelA:p50 | 1.0e-3 | NIK mediated protein degradation | nM^-1^.min^-1^ | cytoplasm |  |
| 117 | RelB:p50:IkBa 🡺  RelB:p50 + IkBa | 8.4e-3 | dissociation | min^-1^ | cytoplasm& nucleus | **Group XXV** |
| 120 | RelB*:p50:IkBa 🡺  RelB*:p50 + IkBa | 8.4e-2 | dissociation | min^-1^ | cytoplasm& nucleus |  |
| 133 | RelB:p50:IkBa 🡺  RelB:p50 | 2e-4 | constitutive protein degradation | min^-1^ | cytoplasm& nucleus |  |
| 136 | RelB*:p50:IkBa 🡺  RelB*:p50 | 2e-4 | constitutive protein degradation | min^-1^ | cytoplasm& nucleus |  |
| 163 | RelB:p50:IkBa + NEMO 🡺RelB:p50 | 1.4e-3 | NEMO mediated protein degradation | nM^-1^.min^-1^ | cytoplasm |  |
| 165 | RelB*:p50:IkBa + NEMO 🡺RelB*:p50 | 1.4e-3 | NEMO mediated protein degradation | nM^-1^.min^-1^ | cytoplasm |  |
| 118 | RelB:p50:IkBe 🡺  RelB:p50 + IkBe | 8.4e-3 | dissociation | min^-1^ | cytoplasm& nucleus | **Group XXVI** |
| 121 | RelB*:p50:IkBe 🡺  RelB*:p50 + IkBe | 8.4e-2 | dissociation | min^-1^ | cytoplasm& nucleus |  |
| 134 | RelB:p50:IkBe 🡺RelB:p50 | 2e-4 | constitutive protein degradation | min^-1^ | cytoplasm& nucleus |  |
| 137 | RelB*:p50:IkBe 🡺  RelB*:p50 | 2e-4 | constitutive protein degradation | min^-1^ | cytoplasm& nucleus |  |
| 164 | RelB:p50:IkBe + NEMO 🡺RelB:p50 | 9.0e-4 | NEMO mediated protein degradation | nM^-1^.min^-1^ | cytoplasm |  |
| 166 | RelB*:p50:IkBe + NEMO 🡺RelB*:p50 | 9.0e-4 | NEMO mediated protein degradation | nM^-1^.min^-1^ | cytoplasm |  |
| 141 | RelA:p50:IkBa 🡺 IkBa | 2e-4 | constitutive protein degradation | min^-1^ | cytoplasm& nucleus | **Group XXVII** |
| 142 | RelA:p50:IkBb 🡺 IkBb | 2e-4 | constitutive protein degradation | min^-1^ | -do- |  |
| 143 | RelA:p50:IkBe 🡺 IkBe | 2e-4 | constitutive protein degradation | min^-1^ | -do- |  |
| 149 | RelB:p50:IkBa 🡺 IkBa | 2e-4 | constitutive protein degradation | min^-1^ | -do- |  |
| 150 | RelB:p50:IkBe 🡺 IkBe | 2e-4 | constitutive protein degradation | min^-1^ | -do- |  |
| 152 | RelB*:p50:IkBa 🡺IkBa | 2e-4 | constitutive protein degradation | min^-1^ | -do- |  |
| 153 | RelB*:p50:IkBe 🡺IkBe | 2e-4 | constitutive protein degradation | min^-1^ | -do- |  |
| 113 | RelA:p52:IkBa 🡺 RelA:p52 + IkBa | 8.4e-3 | dissociation | min^-1^ | cytoplasm& nucleus | * |
| 114 | RelA:p52:IkBb 🡺 RelA:p52 + IkBb | 3.4e-2 | dissociation | min^-1^ | -do- |  |
| 115 | RelA:p52:IkBe 🡺 RelA:p52 + IkBe | 8.4e-3 | dissociation | min^-1^ | -do- |  |
| 116 | RelA:p52:IkBd 🡺 RelA:p52 + IkBd | 1.7e-2 | dissociation | min^-1^ | -do- |  |
| 119 | RelB:p50:IkBd 🡺  RelB:p50 + IkBd | 1.4e-3 | dissociation | min^-1^ | cytoplasm& nucleus | * |
| 122 | RelB*:p50:IkBd 🡺  RelB*:p50 + IkbBd | 1.4e-3 | dissociation | min^-1^ | cytoplasm& nucleus |  |
| 123 | RelB:p52:IkBd 🡺  RelB:p52 + IkBd | 1.4e-3 | dissociation | min^-1^ |  |  |
| 124 | RelB*:p52:IkBd 🡺  RelB*:p52 + IkbBd | 1.4e-3 | dissociation | min^-1^ |  |  |
| 135 | RelB:p50:IkBd 🡺  RelB:p50 | 2e-4 | constitutive protein degradation | min^-1^ | cytoplasm& nucleus | * |
| 138 | RelB*:p50:IkBd 🡺 RelB*:p50 | 2e-4 | constitutive protein degradation | min^-1^ | cytoplasm& nucleus |  |
| 139 | RelB:p50:IkBd 🡺  RelB:p52 | 2e-4 | constitutive protein degradation | min^-1^ | cytoplasm& nucleus |  |
| 140 | RelB*:p50:IkBd 🡺 RelB*:p52 | 2e-4 | constitutive protein degradation | min^-1^ | cytoplasm& nucleus |  |
| 129 | RelA:p52:IkBa 🡺 RelA:p52 | 2e-4 | constitutive protein degradation | min^-1^ | cytoplasm& nucleus | * |
| 130 | RelA:p52:IkBb 🡺 RelA:p52 | 2e-4 | constitutive protein degradation | min^-1^ | cytoplasm& nucleus |  |
| 131 | RelA:p52:IkBe 🡺 RelA:p52 | 2e-4 | constitutive protein degradation | min^-1^ | cytoplasm& nucleus |  |
| 132 | RelA:p52:IkBd 🡺 RelA:p52 | 2e-4 | constitutive protein degradation | min^-1^ | cytoplasm& nucleus |  |
| 145 | RelA:p52:IkBa 🡺 IkBa | 2e-4 | constitutive protein degradation | min^-1^ | cytoplasm& nucleus | * |
| 146 | RelA:p52:IkBb 🡺 IkBb | 2e-4 | constitutive protein degradation | min^-1^ | cytoplasm& nucleus |  |
| 147 | RelA:p52:IkBe 🡺 IkBe | 2e-4 | constitutive protein degradation | min^-1^ | cytoplasm& nucleus |  |
| 148 | RelA:p52:IkBd 🡺 IkBd | 2e-4 | constitutive protein degradation | min^-1^ | cytoplasm& nucleus |  |
| 144 | RelA:p50:IkBd 🡺 IkBd | 2e-4 | constitutive protein degradation | min^-1^ | cytoplasm& nucleus | * |
| 151 | RelB:p50:IkBd 🡺IkBd | 2e-4 | constitutive protein degradation | min^-1^ | cytoplasm& nucleus |  |
| 154 | RelB*:p50:IkBd 🡺 IkBd | 2e-4 | constitutive protein degradation | min^-1^ | cytoplasm& nucleus |  |
| 155 | RelB:p50:IkBd 🡺IkBd | 2e-4 | constitutive protein degradation | min^-1^ | cytoplasm& nucleus |  |
| 156 | RelB*:p50:IkBd 🡺 IkBd | 2e-4 | constitutive protein degradation | min^-1^ | cytoplasm& nucleus |  |
| 160 | RelA:p52:IkBa + NEMO 🡺RelA:p52 | 1.4e-3 | NEMO mediated protein degradation | nM^-1^.min^-1^ | cytoplasm | * |
| 161 | RelA:p52:IkBb + NEMO 🡺RelA:p52 | 4.5e-4 | NEMO mediated protein degradation | nM^-1^.min^-1^ | cytoplasm |  |
| 162 | RelA:p52:IkBe + NEMO 🡺RelA:p52 | 9.0e-4 | NEMO mediated protein degradation | nM^-1^.min^-1^ | cytoplasm |  |
| 045 | IkBa 🡺 IkBan | 6.0e-2 | Nuclear import | min^-1^ | --- | **Group XXVIII** |
| 046 | IkBb 🡺 IkBbn | 9.0e-3 | Nuclear import | min^-1^ | --- | **Group XXIX** |
| 047 | IkBe 🡺 IkBen | 4.5e-2 | Nuclear import | min^-1^ | --- | **Group XXX** |
| 049 | IkBan 🡺 IkBa | 1.2e-2 | Nuclear export | min^-1^ | --- | **Group XXXI** |
| 050 | IkBbn 🡺 IkBb | 1.2e-2 | Nuclear export | min^-1^ | --- | **Group XXXII** |
| 051 | IkBen 🡺 IkBe | 1.2e-2 | Nuclear export | min^-1^ | --- | **Group XXXIII** |
| 048 | IkBd 🡺 IkBdn | 4.5e-2 | Nuclear import | min^-1^ | --- | ***** |
| 052 | IkBdn 🡺 IkBd | 1.2e-2 | Nuclear export | min^-1^ | --- |  |
| 069 | RelA:p50 🡺 RelA:p50n | 5.4 | nuclear import | min^-1^ | --- | **Group XXXIV** |
| 071 | RelB:p50 🡺 RelB:p50n | 5.4 | nuclear import | min^-1^ | --- | **Group XXXV** |
| 074 | RelB*:p50 🡺 RelB*:p50n | 5.4 | nuclear import | min^-1^ | --- |  |
| 070 | RelA:p52 🡺 RelA:p52n | 5.4 | nuclear import | min^-1^ | --- | ***** |
| 072 | RelB:p52 🡺 RelB:p52n | 5.4 | nuclear import | min^-1^ | --- |  |
| 076 | RelB*:p52 🡺 RelB*:p52n | 5.4 | nuclear import | min^-1^ | --- |  |
| 073 | RelB:p100 🡺 RelB:p100n | 4.8e-3 | nuclear import | min^-1^ | --- |  |
| 075 | RelB*:p100🡺 RelB*:p100n | 4.8e-3 | nuclear import | min^-1^ | --- |  |
|  |  |  |  |  |  |  |
| 173 | RelA:p50:IkBa 🡺 RelA:p50:IkBan | 2.7e-1 | nuclear import | min^-1^ | --- | **Group XXXVI** |
| 174 | RelA:p50:IkBb 🡺 RelA:p50:IkBbn | 2.7e-2 | nuclear import | min^-1^ | --- | **Group XXXVII** |
| 175 | RelA:p50:IkBe 🡺 RelA:p50:IkBen | 1.3e-1 | nuclear import | min^-1^ | --- | **Group XXXVIII** |
| 181 | RelB:p50:IkBa 🡺 RelB:p50:IkBan | 2.8e-1 | nuclear import | min^-1^ | --- | **Group XXXIX** |
| 184 | RelB*:p50:IkBa 🡺 RelB*:p50:IkBan | 2.8e-1 | nuclear import | min^-1^ | --- |  |
| 182 | RelB:p50:IkBe 🡺 RelB:p50:IkBen | 1.4e-1 | nuclear import | min^-1^ | --- | **Group XL** |
| 185 | RelB*:p50:IkBe 🡺 RelB*:p50:IkBen | 1.4e-1 | nuclear import | min^-1^ | --- |  |
| 176 | RelA:p50:IkBd 🡺 RelA:p50:IkBdn | 2.7e-1 | nuclear import | min^-1^ | --- | ***** |
| 177 | RelA:p52:IkBa 🡺 RelA:p52:IkBan | 2.7e-1 | nuclear import | min^-1^ | --- |  |
| 178 | RelA:p52:IkBb 🡺 RelA:p52:IkBbn | 2.7e-2 | nuclear import | min^-1^ | --- |  |
| 179 | RelA:p52:IkBe 🡺 RelA:p52:IkBen | 1.3e-1 | nuclear import | min^-1^ | --- |  |
| 180 | RelA:p52:IkBd 🡺  RelA:p52:IkBdn | 2.7e-1 | nuclear import | min^-1^ | --- |  |
| 183 | RelB:p50:IkBd 🡺 RelB:p50:IkBdn | 2.8e-2 | nuclear import | min^-1^ | --- |  |
| 186 | RelB*:p50:IkBd 🡺 RelB*:p50:IkBdn | 2.8e-2 | nuclear import | min^-1^ | --- |  |
| 187 | RelB:p52:IkBd 🡺 RelB:p52:IkBdn | 2.8e-2 | nuclear import | min^-1^ | --- |  |
| 188 | RelB*:p52:IkBd 🡺 RelB*:p52:IkBdn | 2.8e-2 | nuclear import | min^-1^ | --- |  |
| 077 | RelA:p50n🡺 RelA:p50 | 4.8e-3 | nuclear export | min^-1^ | --- | **Group XLI** |
| 079 | RelB:p50n🡺 RelB:p50 | 4.8e-3 | nuclear export | min^-1^ | --- | **Group XLII** |
| 082 | RelB*:p50n 🡺 RelB*:p50 | 4.8e-3 | nuclear export | min^-1^ | --- |  |
| 078 | RelA:p52n 🡺 RelA:p52 | 4.8e-3 | nuclear export | min^-1^ | --- | ***** |
| 080 | RelB:p52n 🡺 RelB:p52 | 4.8e-3 | nuclear export | min^-1^ | --- |  |
| 084 | RelB*:p52n 🡺 RelB*:p52 | 4.8e-3 | nuclear export | min^-1^ | --- |  |
| 081 | RelB:p100n 🡺 RelB:p100 | 4.8e-3 | nuclear export | min^-1^ | --- |  |
| 083 | RelB*:p100n 🡺 RelB*:p100 | 4.8e-3 | nuclear export | min^-1^ | --- |  |
| 189 | RelA:p50:IkBan 🡺 RelA:p50:IkBa | 8.3e-1 | nuclear export | min^-1^ | --- | **Group XLIII** |
| 190 | RelA:p50:IkBbn 🡺 RelA:p50:IkBb | 4.1e-1 | nuclear export | min^-1^ | --- | **Group XLIV** |
| 191 | RelA:p50:IkBen 🡺 RelA:p50:IkBe | 4.1e-1 | nuclear export | min^-1^ | --- | **Group XLV** |
| 197 | RelB:p50:IkBan 🡺 RelB:p50:IkBa | 8.4e-1 | nuclear export | min^-1^ | --- | **Group XLVI** |
| 200 | RelB*:p50:IkBan 🡺 RelB*:p50:IkBa | 8.4e-1 | nuclear export | min^-1^ | --- |  |
| 198 | RelB:p50:IkBen 🡺 RelB:p50:IkBe | 4.2e-1 | nuclear export | min^-1^ | --- | **Group XLVII** |
| 201 | RelB*:p50:IkBen 🡺 RelB*:p50:IkBe | 4.2e-1 | nuclear export | min^-1^ | --- |  |
| 192 | RelA:p50:IkBdn 🡺 RelA:p50:IkBd | 4.1e-1 | Nuclear export | min^-1^ | --- | ***** |
| 193 | RelA:p52:IkBan 🡺 RelA:p52:IkBa | 8.3e-1 | nuclear export | min^-1^ | --- |  |
| 194 | RelA:p52:IkBbn 🡺 RelA:p52:IkBb | 4.1e-1 | nuclear export | min^-1^ | --- |  |
| 195 | RelA:p52:IkBen 🡺 RelA:p52:IkBe | 4.1e-1 | nuclear export | min^-1^ | --- |  |
| 196 | RelA:p52:IkBdn 🡺  RelA:p52:IkBd | 4.1e-1 | nuclear export | min^-1^ | --- |  |
| 168 | RelA:p52:IkBd + NIK-IKK1 🡺RelA:p52 | 1.0e-3 | NIK mediated protein degradation | nM^-1^.min^-1^ | cytoplasm | ***** |
| 169 | RelB:p50:IkBd + NIK-IKK1 🡺RelB:p50 | 1.0e-3 | NIK mediated protein degradation | nM^-1^.min^-1^ | cytoplasm |  |
| 170 | RelB*:p50:IkBd + NIK-IKK1 🡺RelB*:p50 | 1.0e-3 | NIK mediated protein degradation | nM^-1^.min^-1^ | cytoplasm |  |
| 171 | RelB:p52:IkBd + NIK-IKK1 🡺RelB:p52 | 1.0e-3 | NIK mediated protein degradation | nM^-1^.min^-1^ | cytoplasm |  |
| 172 | RelB*:p52:IkBd + NIK-IKK1 🡺RelB*:p52 | 1.0e-3 | NIK mediated protein degradation | nM^-1^.min^-1^ | cytoplasm |  |
| 199 | RelB:p50:IkBd 🡺 RelB:p50:IkBdn | 4.2e-1 | nuclear export | min^-1^ | --- | ***** |
| 202 | RelB*:p50:IkBdn 🡺 RelB*:p50:IkBd | 4.2e-1 | nuclear export | min^-1^ | --- |  |
| 203 | RelB:p52:IkBdn 🡺 RelB:p52:IkBd | 4.2e-1 | nuclear export | min^-1^ | --- |  |
| 204 | RelB*:p52:IkBdn 🡺 RelB*:p52:IkBd | 4.2e-1 | nuclear export | min^-1^ | --- |  |
| 205 | p100 + p100 🡺IkBd | 8.1e-2 | p100 association | nM^-1^.min^-1^ | cytoplasm | ***** |
| 206 | IkBd 🡺 p100 + p100 | 1.2e-5 | IκBδ dissociation |  | cytoplasm |  |
| 207 | p100 + NIK-IKK1 🡺 p52 | 2.1e-2 | p100 processing through NIK | nM^-1^.min^-1^ | cytoplasm |  |
| 039 | p52 🡺 | 5.7e-3 | Protein decradation | min^-1^ | cytoplasm & nucleus |  |
| 208 | RelB🡺 RelB* | 1.2e-2 | RelB modification | min^-1^ | cytoplasm | **Group XLVIII** |
| 209 | RelB:p50 🡺 RelB*:p50 | 1.2e-2 | RelB modification | min^-1^ | cytoplasm |  |
| 211 | IkBa: RelB:p50 🡺 IkBa:RelB*:p50 | 1.2e-2 | RelB modification | min^-1^ | cytoplasm |  |
| 212 | IkBe: RelB:p50 🡺 IkBe:RelB*:p50 | 1.2e-2 | RelB modification | min^-1^ | cytoplasm |  |
| 213 | IkBd: RelB:p50 🡺 IkBd:RelB*:p50 | 1.2e-2 | RelB modification | min^-1^ | cytoplasm | ***** |
| 214 | IkBd: RelB:p52 🡺 IkBd:RelB*:p52 | 1.2e-2 | RelB modification | min^-1^ | cytoplasm |  |
| 210 | RelB:p50 🡺 RelB*:p50 | 1.2e-2 | RelB modification | min^-1^ | cytoplasm |  |
